# Long Non-Coding RNA and Alternative Splicing Modulations in Parkinson's Leukocytes Identified by RNA Sequencing

**DOI:** 10.1371/journal.pcbi.1003517

**Published:** 2014-03-20

**Authors:** Lilach Soreq, Alessandro Guffanti, Nathan Salomonis, Alon Simchovitz, Zvi Israel, Hagai Bergman, Hermona Soreq

**Affiliations:** 1 Department of Medical Neurobiology, IMRIC, The Hebrew University-Hadassah Medical School, Jerusalem, Israel; 2 Department of Biological Chemistry, The Life Sciences Institute, The Hebrew University of Jerusalem, Jerusalem, Israel; 3 Genomnia srl, Lainate, Milan, Italy; 4 Department of Pediatrics, Division of Biomedical Informatics, Cincinnati Children's Hospital Medical Center, Cincinnati, Ohio, United States of America; 5 The Center for Functional and Restorative Neurosurgery, Department of Neurosurgery, Hadassah University Hospital, Jerusalem, Israel; 6 The Edmond and Lily Safra Center for Brain Sciences (ELSC), The Hebrew University of Jerusalem, Jerusalem, Israel; Accelrys, United States of America

## Abstract

The continuously prolonged human lifespan is accompanied by increase in neurodegenerative diseases incidence, calling for the development of inexpensive blood-based diagnostics. Analyzing blood cell transcripts by RNA-Seq is a robust means to identify novel biomarkers that rapidly becomes a commonplace. However, there is lack of tools to discover novel exons, junctions and splicing events and to precisely and sensitively assess differential splicing through RNA-Seq data analysis and across RNA-Seq platforms. Here, we present a new and comprehensive computational workflow for whole-transcriptome RNA-Seq analysis, using an updated version of the software AltAnalyze, to identify both known and novel high-confidence alternative splicing events, and to integrate them with both protein-domains and microRNA binding annotations. We applied the novel workflow on RNA-Seq data from Parkinson's disease (PD) patients' leukocytes pre- and post- Deep Brain Stimulation (DBS) treatment and compared to healthy controls. Disease-mediated changes included decreased usage of alternative promoters and N-termini, 5′-end variations and mutually-exclusive exons. The PD regulated FUS and HNRNP A/B included prion-like domains regulated regions. We also present here a workflow to identify and analyze long non-coding RNAs (lncRNAs) via RNA-Seq data. We identified reduced lncRNA expression and selective PD-induced changes in 13 of over 6,000 detected leukocyte lncRNAs, four of which were inversely altered post-DBS. These included the U1 spliceosomal lncRNA and RP11-462G22.1, each entailing sequence complementarity to numerous microRNAs. Analysis of RNA-Seq from PD and unaffected controls brains revealed over 7,000 brain-expressed lncRNAs, of which 3,495 were co-expressed in the leukocytes including U1, which showed both leukocyte and brain increases. Furthermore, qRT-PCR validations confirmed these co-increases in PD leukocytes and two brain regions, the amygdala and substantia-nigra, compared to controls. This novel workflow allows deep multi-level inspection of RNA-Seq datasets and provides a comprehensive new resource for understanding disease transcriptome modifications in PD and other neurodegenerative diseases.

## Author Summary

Long non-coding RNAs (lncRNAs) comprise a novel, fascinating class of RNAs with largely unknown biological functions. Parkinson's-disease (PD) is the most frequent motor disorder, and Deep-brain-stimulation (DBS) treatment alleviates the symptoms, but early disease biomarkers are still unknown and new future genetic interference targets are urgently needed. Using RNA-sequencing technology and a novel computational workflow for in-depth exploration of whole-transcriptome RNA-seq datasets, we detected and analyzed lncRNAs in sequenced libraries from PD patients' leukocytes pre and post-treatment and the brain, adding this full profile resource of over 7,000 lncRNAs to the few human tissues-derived lncRNA datasets that are currently available. Our study includes sample-specific database construction, detecting disease-derived changes in known and novel lncRNAs, exons and junctions and predicting corresponding changes in Polyadenylation choices, protein domains and miRNA binding sites. We report widespread transcript structure variations at the splice junction and exons levels, including novel exons and junctions and alteration of lncRNAs followed by experimental validation in PD leukocytes and two PD brain regions compared with controls. Our results suggest lncRNAs involvement in neurodegenerative diseases, and specifically PD. This comprehensive workflow will be of use to the increasing number of laboratories producing RNA-Seq data in a wide range of biomedical studies.

## Introduction

Recent studies have identified conspicuous diversity in large intergenic long non-coding RNAs (lncRNAs) found across many species [1] [2]. LncRNAs are currently defined as transcripts of over 200 nucleotides [3]. Nonetheless, the GENCODE non-coding RNA set, the largest currently lncRNA database, contains currently as much as 136 spliced transcript shorter than 200 bp, and the general and structural annotation of lncRNA overall is still ongoing [4]. LncRNAs may contain open reading frames (ORF), and are often transcribed by RNA polymerase II, spliced and polyadenylated – but do not code for any protein product. LncRNAs are the least well studied among thousands non-coding eukaryotic RNAs that have been discovered so far. While genome-wide expression and evolutionary analyses suggest that some of them play functional roles, their cellular mechanisms of action are still largely unknown [5]. Nonetheless, accumulating evidence suggests that in the nervous system, lncRNA functions span regulating brain evolution and neural development [6] and mediate behavioral and cognitive processes [7]. In Drosophila, the neuronal-expressed CRG lncRNA is involved in regulating locomotion by recruiting RNA polymerase II to the adjacent promoter of the movement-related protein-coding gene CASK, thereby increasing CASK expression [8]. In humans, lncRNAs are involved in neurogenesis, neuropsychiatric disorders [9], cancer (for example, HT19 which is involved in tumor growth) [10]) and in Autism [11] as well as in the neurodegenerative Huntington's [12] and Alzheimer's (AD) diseases [13]. However, the involvement of lncRNAs in the leading neurodegenerative motor disorder worldwide, Parkinson's disease (PD), is still unknown.

PD is the second most common neurodegenerative disease worldwide (after AD) [14], [15], with age being the leading risk factor currently known and no known cure. It affects 1–2% of the population above 65 years of age [16], [17], [18] and is characterized by four cardinal motor symptoms (resting tremor, bradykinesia (“slow movement”), postural instability and akinesia (“lack of movement”) [19] [20]. These appear when most of the brain's dopamine-producing neurons have already been diminished. Most cases are defined as ‘sporadic’ and treatment is aimed at replacing lost DA through adjusting the declining levels of the precursor L-Dopa. The alternative, deep brain stimulation (DBS) treatment allows a significant reduction in the medication dosage while drastically improving motor function in patients. DBS presumably alleviates the disease symptoms by targeting the basal ganglia Sub-Thalamic Nucleus (STN) brain region through yet undefined mechanisms [21].

While the underlying aetiology of sporadic PD remained elusive, genomics studies have implicated several genes in the loss of DA neurons. Mutations in α-synuclein (SNCA), the first gene identified as linked to PD [22], cause early-onset PD, and the SNCA protein product has been identified as a major component of Lewy bodies [23], a morphological pathological hallmark of PD [24]. Mutations in the ubiquitin ligase Parkin (PARK2) that targets proteins for degradation in the proteasome through linkage of ubiquitin molecules cause DA neuron pathology [25] and autosomal recessive Parkinsonism [26]. The DJ-1 (PARK7) protein regulates oxidation–reduction signalling pathways via inducing gene expression [27], inhibiting the formation of SNCA aggregates [28] and limiting dopaminergic cell death in cellular and animal PD models [29]. Mutations in the putative serine threonine kinase LRRK2 (PARK8) cause uncoupling of mitochondria in fibroblast and neuroblastoma cells [30]; and the PTEN-induced protein kinase PINK1 (PARK6), mutations in which cause early-onset PD [31] is believed to be involved in mitophagy [32]. Taken together, these observations suggest that inherited, and possibly acquired impairments in the pathways regulating protein metabolism, oxidative stress and mitochondrial functioning are causally involved in PD emergence. Yet, current medications only improve the disease motor symptoms – but do not provide a cure. Furthermore, identification of the disease in its early stages, before the majority of the dopaminergic neuron population have diminished, is currently impossible.

Transcriptome analysis of peripheral blood is of great interest for clinical research, as differences between samples obtained in a minimally invasive and cost-effective manner can be translated into gene signatures of disease, as well as disease stage, drug response and toxicity [7]. Blood cells interact with most tissues and organs in the human body and their cellular composition provides a reflection of both physiological and pathogenic stimuli, including brain treatment effects [33]. Furthermore, 80% of the genes expressed in peripheral blood cells are shared with other central tissues [12]. While nucleated white blood cells make up the minority of blood cells, they are the most informative. Correspondingly, gene expression differences in peripheral whole blood have been used to determine gene signatures related to both acute myeloid leukemia [8] and neuropsychiatric disorders and Huntington's disease, where significant correlation between blood and brain transcripts was identified [9], [10]. Other neurological diseases for which peripheral blood-based biomarkers have been identified include multiple sclerosis, schizophrenia and Alzheimer's disease [34], [35], [36]. These effects have been specifically attributed to neuronal death, neuronal cell-free RNA expression and well-described neuro-immune modulatory effects [34], [35], [36]. Likewise, we have recently observed parallel changes in microRNAs (miRNAs) and genes predicted as their targets that further underwent splicing changes in PD leukocytes and in PD-relevant brain regions, including the substantia nigra (SN) as well as the frontal lobe) through coupled analysis of small RNA-Seq data and splice junction arrays [37]. These spanned immune, mitochondrial and oxidative stress changes, supporting our microarray identification of interleukin-4 (IL4) related processes in whole blood data from a large early PD cohort [38]. Since the first report of miRNA involvement in PD [39], new findings provide ample evidence for involvement of differentially expressed miRNAs in the PD brain [40] [41] [42] [43] [44] [45] [46]. In differential expression studies of PD patients' leukocytes, we found expression changes that were partially reversed following DBS treatment [47], [48]. Parallel changes were also detected in both the frontal cortex and the caudate-putamen brain areas from PD model mice treated with 1-methyl-4-phenyl-1,2,3,6-tetrahydropyridine (MPTP) neurotoxin. Thus, leukocyte datasets provide useful resource for identifying possible disease biomarkers (which are urgently needed for PD), and for studying PD-related processes in an easily accessible tissue. With the advent of current genomic technologies, new tools are required for linking between different datasets produced from various technologies including different types of microarrays, and RNA-Seq of both long and short molecules.

Other potentially involved regulators of other transcripts are microRNAs (miRNAs), ∼22 nucleotides small non-coding RNAs processed by Drosha and Dicer from larger pri-miRNA molecules (the initial miRNA transcript) and pre-miRNA (i.e the 65–70 nucleotide hairpin) [49]. Binding of mature miRNAs to RNA-induced silencing complexes (RISC) is followed by guidance to target cognate protein-coding mRNAs, largely through identification of ‘seed’ matches (sequences complementary to positions 2–8 of the miRNA) in the 3′ un-translated region. The miRNA-RISC complex then initiates a program for mRNA degradation or block of translation. Of note, miRNAs can operate in tandem, cooperatively, or without an apparent seed sequence match. Since the first report of miRNA involvement in PD [39], new findings provide ample evidence for involvement of differentially expressed miRNAs in the PD brain [40] [41] [42] [43] [44] [45] [46]. Much of these changes are reflected in PD leukocytes, where we recently observed by microarray analyses coupled miRNA-Alternative Splicing (AS) modifications that were modulated by DBS [37]. Thus, leukocyte datasets provide useful resources for studying PD-related processes, and new tools are required for linking between these different datasets.

**Figure 1:**
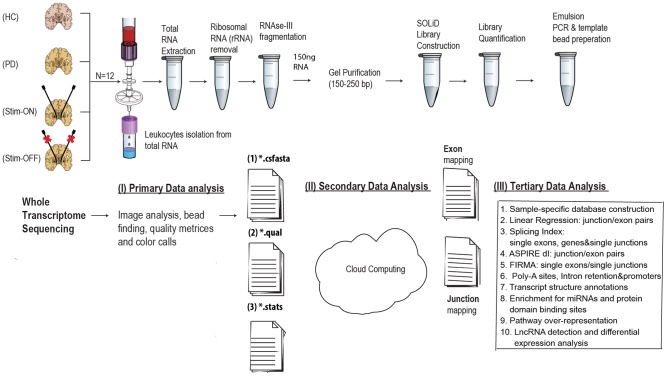
General outline of the RNA-Seq experiment and analysis workflow. RNA was extracted from PD leukocytes pre-DBS, post-DBS on stimulation and following one hour of electrical stimulation cessation (off-Stim). Libraries were constructed for SOLiD whole transcriptome sequencing following ribosomal RNA removal, fragmentation and gel purification. Primary analysis of the 50 nucleotide long reads was followed by mapping of the outcome reads to the human genome through BioScope cloud computation. Tertiary data analysis workflow was developed through a novel RNA-Seq module of AltAnalyze (version 2.0). The analysis steps included: sample-specific database construction followed by detection of known and novel junctions and exons. Four different algorithms were used for measurement of splicing changes from single features (junctions/exons) and feature pairs. These included: linear regression, splicing-index, ASPIRE and FIRMA. Subsequent annotation of the splicing events at the transcript level enabled detection of various annotations including 3′ and 5′ end variations, cassette exons and mutually exclusive exons. Additionally, detection of both promoter region and splicing alternations as well as intron retention events was enabled, and a novel module for poly-A site prediction was incorporated. Enrichment for protein binding domains and miRNA target sites was conducted using z-score and the corresponding databases. Additionally, detection of all the lncRNAs expressed in these samples expressed lncRNAs was conducted through alignment to GENCODE 7 database, and followed by differential expression analysis (EdgeR) and predictions of secondary structure and potential miRNA complementarity predictions.

In our current study, we employed whole-transcriptome RNA sequencing and a newly developed workflow that includes comprehensive RNA-Seq analysis approaches to characterize all of the leukocyte-expressed protein coding transcripts as well as the non-coding class of lncRNAs in PD patients and control volunteers. We identified splicing changes, as well as novel exons and junctions by implementation widespread gene-, exon- and junction-level analyses. We implemented a variety of differential expression and splicing analysis methods including linear regression, Splicing Index (SI), ASPIRE and FIRMA. This enabled an integrated analysis of exons and junctions (both separately and combined), transcript structural variations and functional processes, and allowed the conduction of integrated RNA-Seq analysis of whole transcriptome data. We also implemented a new module that enables identification of known and novel Poly-A sites. Our comprehensive novel RNA-Seq analysis workflow further enabled the identification of specific protein domains translated from sequence regions detected as spliced, as well as potential miRNA binding sites within the detected regions. We applied this vast range of analysis tools on PD leukocytes from PD patients (pre-DBS) and post treatment (post-DBS) while being either on- or off-electrical stimulation, all as compared to matched controls the Amygdala and SN. The experimental and analysis workflow is illustrated under Figure 1. Additionally, we used a publically available junction array datasets to characterize knock out effects of two PD leukocyte modified genes that are involved in additional neuropathologies, both include prion-like domains: FUS and HNRNP A/B [50]. We further analyzed an additional independent RNA-Seq dataset from PD brain samples and compared the PD blood and leukocyte modified lncRNAs to the brain modified ones following the full characterization of PD brain expressed lncRNAs in the external dataset. Experimental quantitative reverse-transcription polymerase chain reaction (qRT-PCR) tests validated exemplary findings both in patients' leukocytes and two brain regions from an additional set of PD brain samples as compared with unaffected control brain samples.

## Results

To deeply delineate splicing modulations and to characterize the full profile of lncRNAs as well as their differential expression in PD, we implemented a novel and comprehensive RNA-Seq analysis workflow (available as version 2.0 of AltAnalyze [51] program). We applied the full analysis workflow on whole-transcriptome RNA-Seq data from blood leukocytes of PD patients pre- and post-DBS treatment in two states: on- and following one hour off electrical stimulation, as well as from age- and gender-matched healthy control (HC) volunteers (Table S1A). Overall, 12 RNA-Seq libraries were produced (of 3 replicates per condition) (all deposited under the Gene Expression Omnibus [52], GEO accession number GSE42608). The total number of read counts per library ranged between 75,174,576 −111,910,462 (Table S1B). Of them, 54%–71% (which were composed of 41,413,564 to 74,845,764 reads, median: 60,933,494 reads, or 67%) were mapped to the human genome (UCSC genome assembly, version 19). The total sequenced read counts did not show statistically significant differences between the different clinical groups (PD patients pre- and post-DBS or healthy control samples). Uniquely aligned exon reads amounted to 62.4%–74.4% of the total number of mapped reads. Overall, 633,054 exons were identified in the RNA-Seq libraries; Reads that were aligned to unique junctions composed 2.4%–3.8% of the total number of aligned reads. Using the newly developed analysis workflow that we present here, we identified as many as 344,009 of them (54%) as novel human exons. The rest (289,045, composing of 46%) were previously annotated in either the EnsEMBL [53] or in the UCSC [54] human genome databases. Similarly, of the 321,808 junctions identified in total in the RNA-Seq libraries, 102,053 (32%) were novel. The rest (219,755, or 68%) were already annotated.

### LncRNAs are widely expressed in PD leukocytes and selectively altered in the disease

**Figure 2:**
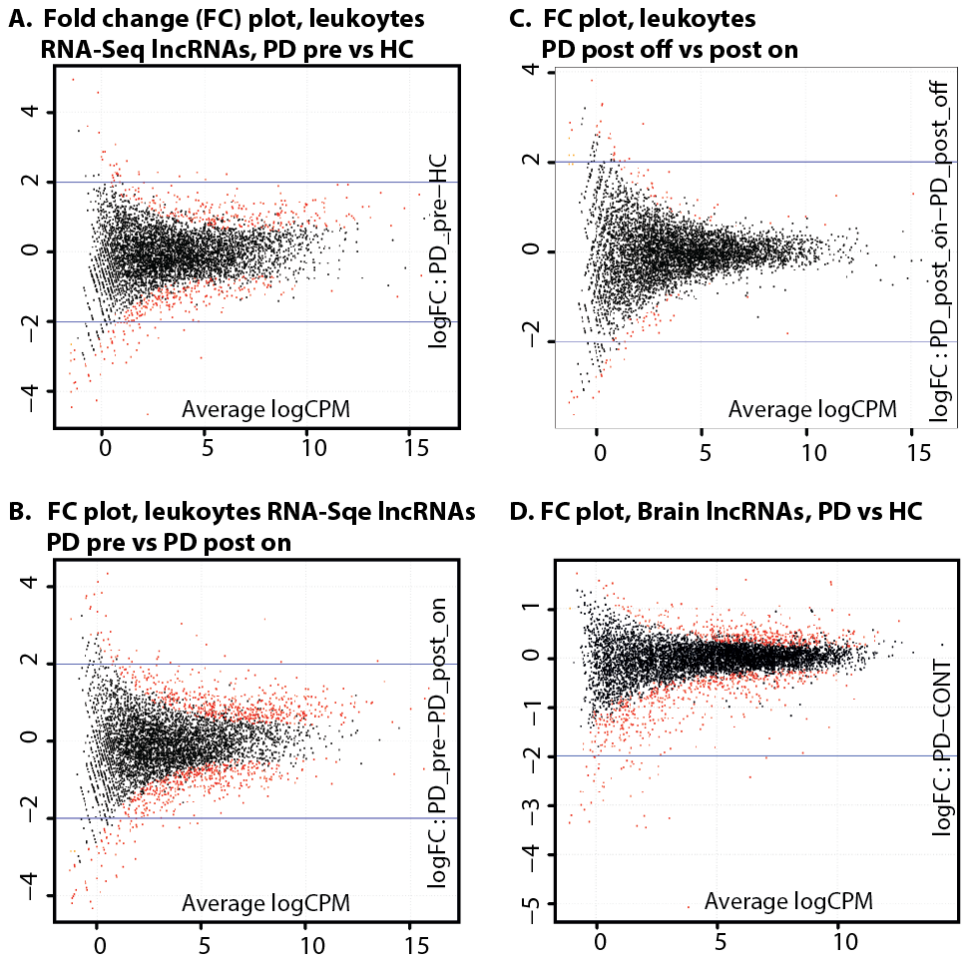
LncRNAs are expressed in leukocytes and exhibit differential expression in PD and following DBS. Over 5,800 lncRNAs were detected as expressed in RNA from PD patient's leukocyte pre- and post-DBS (both on and off electrical stimulation) and in control leukocyte RNA samples through alignment of the RNA-Seq read counts to GENCODE database (A). (B) Differential expression analysis of the detected lncRNAs using common dispersion model and trimmed normalization identified 13 lncRNAs as highly significantly changes (FDR<0.05) in PD patients leukocytes pre-DBS as compared with HC, 8 exhibiting increase and 5 – decrease in PD (C) Differential expression analysis of the post-DBS leukocyte samples compared to the pre-DBS state of the same patients leukocytes detected 18 DBS-modified lncRNAs (4 up- and 14 down-regulated), four of which also changed in the disease pre- treatment (color marked). Note inverse directions of change post-DBS as compared with pre-DBS in all the overlapping lncRNAs (D) Real-time quantitative PCR (qPCR) validated the disease-induced patients leukocytes increases of spliceosomal lncRNA U1 (left), in the lncRNA RP11-79P5 (middle) and in RP11-462G22 (right) in patients' leukocytes. (E–F): Parallel validations in two brain region samples from PD patients and age-matched male controls from the Netherland's Brain Bank (NBB): the SN (E) and Amygdala (F). In all sections, the Y axis is averaged delta Ct fold change, and * marks t-test p<0.05. Leukocyte reference gene: RPL19, brain reference gene: TUBB3.

So far, only a few RNA-Seq studies have detected or analyzed lncRNAs [55]. We took advent of our next generation RNA-Seq data to identify all of the leukocyte-expressed lncRNAs and to search for differentially expressed lncRNAs in disease- and post-surgical treatment state. For this purpose, we mapped all of the sequenced reads detected in leukocytes from PD patients pre- and post-DBS and from matched healthy control (HC) volunteers against the largest currently available lncRNA database, the GENCODE database version 7 [56]. This database consists of reconstructed transcript models using Exonerate [57] and Scripture [58], and is based on the high-depth transcriptomic data from 16 human tissues made publicly available from Illumina [56]. The current version of the GENECODE database covered 14,880 partially identified lncRNAs based on their chromatin signatures or position relative to protein coding genes. Alignment of the RNA-Seq leukocyte data to GENCODE revealed an average of 6,209 leukocyte expressed lncRNAs (across all the leukocyte sequenced libraries), of them as many as 5,862 were present in all the libraries. Generally, PD patient leukocytes at all clinical stages (either pre- or post- DBS treatment, and comparing on- and off- electrical stimulation) contained less lncRNAs than in HC (Figure 2A).

**Figure 3:**
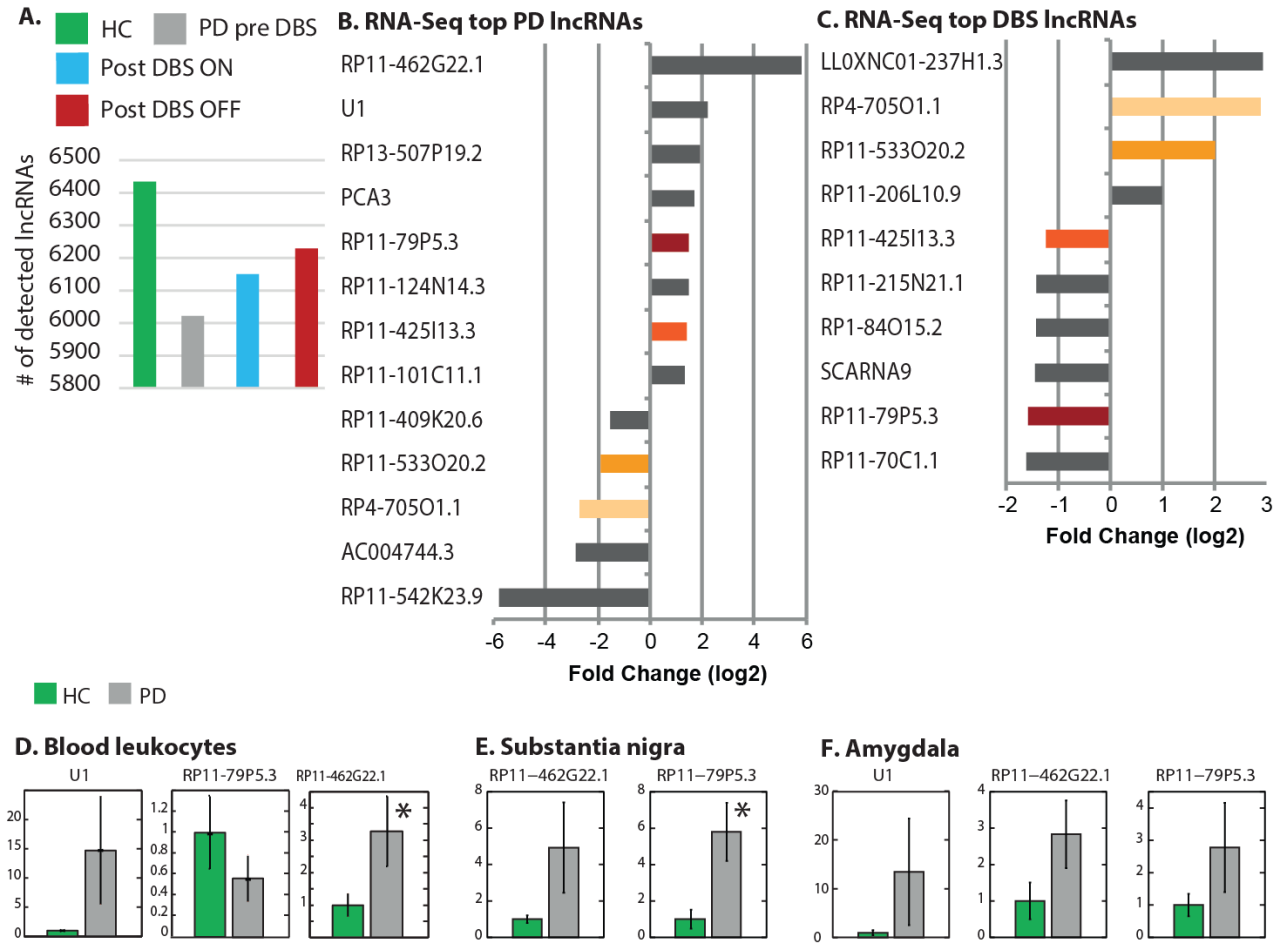
Dispersion plots show parallel change patterns in PD leukocytes and brain tissues. (A) The fold change smear plot of the lncRNAs detected in PD patients' blood leukocytes as compared with HC using tagwise dispersion is slightly skewed towards positive log fold change indicating a general up-regulation trend in PD leukocytes (x axis: average log count per millions of RNA-Seq reads, y axis: log fold change PD compared to HC). The top most differentially expressed lncRNAs (n = 595, uncorrected p<0.05) are highlighted in red. (B) Fold change smear plot for lncRNAs in blood leukocytes following Deep Brain Stimulation (DBS) treatment as compared with the pre-DBS leukocyte RNA from the same PD patients show the 663 differentially expressed ones (uncorrected p<0.05) exhibited massive down-regulation of 428 lncRNAs. (C) A short one hour of electrical stimulation cessation post-DBS induced very mild lncRNA modifications (n = 110 differentially expressed lncRNAs, uncorrected p<0.05), equally down- and up- regulated under electrical stimulation cessation (n = 55 in each group). (D) Of over 7,000 lncRNAs detected through PD brain RNA-Seq data analysis, 964 were altered in PD brain samples compared with unaffected brains (red colored), 242 of these passed FDR correction. A global trend of down-regulation of the brain lncRNAs under PD is clearly evident.

We applied differential expression analysis of all the lncRNAs that were expressed in all the libraries using EdgeR [59], [60]. The analysis used an over-dispersed Poisson model (to account for both biological and technical variability) and Empirical Bayes methods (to moderate the degree of over-dispersion across transcripts and improve the reliability of the results). The normalization approach employed an empirical strategy that equates the overall expression levels of genes between samples under the assumption that the majority of them are not differentially expressed [59], [60]. Overall, the analysis revealed 596 PD leukocytes modified lncRNAs (p<0.05). Briefly, the fold change tagwise dispersion plot of the lncRNAs detected in PD patient leukocytes was slightly skewed towards positive log fold change, indicating a general up-regulation trend in PD leukocytes (Smear plot is illustrated under Figure 3A). The biological coefficient of variation (BCV) and multidimensional scaling clustering (MDS) plots are given under Figure S1. Among all of the PD leukocyte altered lncRNAs compared to HC (with uncorrected p<0.05), 13 passed FDR correction (FDR<0.05, Table 1). Overall, 11 of them were annotated by GENCODE 7.0 as novel entities. Those were well supported by locus-specific transcript evidence or evidence from a paralogous or orthologous locus while not being currently represented in the two databases of HUGO Gene Nomenclature Committee (HGNC) database [61] and RefSeq [62]. Two of the altered lncRNAs were annotated as known two additional databases, and overall −8 of the altered lncRNAs were on the sense and 5 on the antisense strand.

**Table 1:** LncRNAs differentially expressed in PD leukocytes.

N	Gene Name	Ensembl Transcript ID	Fold change (Log)	RNA-Seq FDR	Chr.	Avg. CPM, leukocyte RNA-Seq data	Avg. CPM, brain RNA Seq data (Bold: p<0.05)
1	RP11-101C11.1	ENST00000451250	1.34	0.007	1	168.75 (119.85)	88.24
2	RP11-409K20.6	ENST00000414926	−1.56	0.01	9	52.3 (37.46)	8.01
3	U1*^,^ #	ENST00000415386	2.19	0.01	1	17.25 (11.4)	Absent
4	RP11-425I13.3	ENST00000512692	1.38	0.01	4	605.16 (426.69)	575.49
5	RP11-124N14.3	ENST00000456355	1.45	0.01	10	2,459.91 (1,754.36)	Absent
**6**	**RP11-79P5.3** #	**ENST00000505955**	**1.51**	**0.02**	**5**	**111 .41(80.17)**	**4.79**
7	RP4-705O1.1	ENST00000412672	−2.73	0.02	20	11.3 (8.42)	Absent
**8**	**AC004744.3**	**ENST00000447057**	**−2.88**	**0.02**	**7**	**8.41 (5.97)**	**339.01**
9	RP11-462G22.1*^,^ #	ENST00000513681	5.77	0.02	4	2.25 (1.56)	Absent
10	RP11-533O20.2	ENST00000412701	−1.93	0.02	6	67.16 (50)	Absent
11	RP11-542K23.9	ENST00000412262	−5.82	0.02	9	2.41 (1.41)	Absent
**12**	**RP13-507P19.2**	**ENST00000428272**	**1.90**	**0.02**	**12**	**17.25 (12.25)**	**68.74**
13	PCA3	ENST00000412654	1.71	0.04	9	33.08 (23.14)	6.74

Differentially expressed lncRNAs in PD leukocytes compared to HC. Among all the leukocyte RNA-Seq detected lncRNAs, 13 exhibited differential expression in PD patients compared to matched control volunteers (FDR<0.05). Of these, 8 exhibited disease-mediated decrease, and 5- increase. For each lncRNA, given are is ensEMBL transcript ID, log fold change in PD (pre-DBS) compared to HC, FDR and chromosomal location, as well as the average count read, and count per million (CPM) in the leukocyte sequenced libraries. The brain CPM values are given as well where detected. The predicted targeted miRNAs for the 2 PD leukocyte modified possible competitive endogenous lncRNAs (ceRNAs) - the U1 small nuclear ribonucleic particle (snRNP) spliceosome component and RP11-462G22.1 (marked with *) are: for U1 - hsa-miR-188-3p, hsa-miR-512-3p, hsa-miR-125b-5p, hsa-miR-1281, hsa-miR-5091, hsa-miR-4254, hsa-miR-125a-5p, hsa-miR-4319, and for RP11-462G22.1 - hsa-miR-5093, hsa-miR-877-3p, hsa-miR-27b-5p, hsa-miR-4695-5p, hsa-miR-539-5p, hsa-miR-25-5p, hsa-miR-4730, hsa-miR-4727-3p, hsa-miR-1277-5p, hsa-miR-4307, hsa-miR-29b-2-5p, hsa-miR-3142, hsa-miR-4446-5p, hsa-miR-548t-5p, hsa-miR-3922-5p, hsa-miR-769-5p, hsa-miR-3662, hsa-miR-650, hsa-miR-139-5p, hsa-miR-4494, hsa-miR-3612. Bold: lncRNAs that were also detected as altered in RNA-Seq data from PD patients' brain samples as compared with HC brains.

# : validated by qRT-PCR in both PD leukocytes and 2 brain regions (Amygdala and Substantia Nigra) of PD and unaffected individuals (the Netherlands Brain Bank).

The majority of the lncRNAs found to be differentially expressed in PD leukocytes belong to the novel multi-exon RNA processing (RP-) lncRNAs family, with four of them showing locus conservation with zebra fish and one with mouse [63]. One of the disease leukocyte-altered lncRNAs (RP11-124N14.3, transcript name RP11-124N14.3-001) showed high abundance (with an average count level of 2,460 in all the sequenced libraries), whereas the rest of the lncRNAs found to be differentially expressed in PD showed middle to low abundance levels (hundreds-to numerous counts; Table 1). The PD leukocyte-altered lncRNAs further had different transcript size, some shorter (for example, RP11-533O20.2 of 2 exons and non-conventional length for lncRNA of 161 nucleotides (nt)), some with middle length (e.g, RP11-462G22.1 – of 879 nt) and some longer (e.g U1, with length of 1,548 nt and RP4-705O1.1 – 1,518 nt). Notably, one of the DBS up-regulated lncRNAs, RP11-120K24.2, was reported to be up-regulated in the brain of Autism disorder patients [64].

### Real-time PCR validation of lncRNAs altered in both PD blood leukocyte and brain

The disease-mediated increase observed by RNA-Seq analysis in U1 and RP11-462G22.1 was faithfully validated by real-time RT-PCR in the PD leukocytes (Figure 2D, one-tailed t-test p = 0.049 for RP11-462G22.1 and RPL19 as reference gene, non-significant for U1; details under Methods), which further raised the question of the relevance of the observed leukocyte alterations to the PD brain degenerative process. Next, quantitative RT-PCR (qRT-PCR) in two PD-related brain tissues, the Amygdala and the SN, validated the leukocyte PD observed increase of potential ceRNA RP11-462G22.1 (Figures 2E and 2F, two-way ANOVA p = 0.049, TUBB3 served as a reference gene). Validation in both brain regions by qRT-PCR of the disease RNA-Seq detected differential expression of the lncRNA RP11-79P5.3 (LncBTF3-4, which is conserved in the zebra fish) have confirmed its observed PD leukocyte up-regulation in PD brains as well (Figure 2E and 2F; two-tailed t-test p = 0.03, TUBB3 served as a reference gene). The increase of U1, the second leukocyte altered lncRNA which was detected as altered in PD leukocytes through leukocyte RNA-Seq analysis and was predicted to target numerous miRNAs, was confirmed by qRT-PCR in PD leukocytes (Figure 2D). In the brain, it was detected by PCR only in the Amygdala (and not in the SN), where it also exhibited elevation under PD as in PD blood leukocytes (Figures 2D and 2F).

### DBS modulates lncRNAs expression in blood leukocytes

The DBS treatment induced differential expression changes in lncRNAs. (Figure 3B). Overall 663 lncRNAs (uncorrected p<0.05) were modified post-DBS as compared with the pre-DBS (disease) state of the same patients exhibiting mainly post- treatment down regulation (Smear plot is illustrated under Figure 3B). While 428 lncRNAs decreased post- compared to pre-DBS, only 235 increased post-treatment. Thus, overall, the DBS expressed a wider effect on lncRNAs as compared with the disease. The treatment induced opposite global direction of change compared to the disease, which mainly induced leukocyte lncRNA increases. The full list of differentially expressed lncRNAs with RNA-Seq count values is given under Table S2 (MDS and BCV plots are illustrated under Figure S2). Of the total number of DBS modified lncRNAs, 18 passed FDR correction (Figure 2C). Of the highly significant treatment-altered lncRNAs (FDR<0.05, Table S2), 9 were on the sense and 9 on the antisense strands. Of the DBS-modified lncRNAs, 14 showed a decrease and only 4 were increased post- as compared with pre-DBS (Figure 2C). Four of the lncRNAs that were modified post- as compared to pre-DBS in the blood leukocytes were among the top PD leukocyte modified lncRNAs (as shown in Figures 2B and 2C, color highlighted): RP4-705O1.1, RP11-533O10.2, RP11-425I13.3 and RP11-79P5.3 (of which disease increase was validated by qRT-PCR in patients pre-DBS compared to HC). All the disease- and treatment- shared lncRNAs showed inverse direction of expression changes post- as compared with pre-DBS.

The short one hour electrical stimulation cessation (OFF-stimulus) induced very mild alteration of lncRNA expression in the patients' leukocytes, as it did not induce any lncRNA differential expression that passed FDR correction (Fold change plot under Figure 3C, MDS and BCV plot under Figure S3). Nonetheless, 110 LncRNAs showed uncorrected significance level of p<0.05 (Table S2C), completely balances in terms of down- and up-regulation – 55 lncRNAs exhibited leukocyte decrease upon stimulation cessation, and 55 increase. These included two molecules that were annotated by GENCODE as putative lncRNAs - transcripts that contain 3 or fewer exons and are supported by 1 or 2 Expressed Sequence Tags (ESTs), but not 3.

### Characterization of brain-expressed lncRNAs that exhibit PD-induced alternations and overlap with PD leukocyte altered lncRNAs

To further challenge the significance of PD leukocyte modified lncRNAs, we analyzed an additional, independent recently released RNA-Seq dataset from post mortem brain samples of PD patients and healthy donors (Array expression accession number E-GEOD-40710). This dataset was composed of the 3′ UTR of polyadenylated mRNA sequencing data (PA-Seq) of transcripts from cortical tissue samples of PD patients and unaffected controls [65]. We selected 6 PD and 6 age- and gender- leukocyte-matched (males) non-affected samples from this dataset for analysis. MDS plot (illustrated under Figure S4) revealed that two of the unaffected control samples as potential outliers, and therefore we excluded these samples from further analysis, and analyzed for disease differential expression only the remaining 6 PD and 4 unaffected individual samples from the external PD brain RNA-Seq dataset (count values are given under Table S3A). Overall, a larger number of lncRNAs were expressed in the brain as compared with blood leukocytes (n = 7,189; Table S3A). Of these, 3,495 lncRNAs were also among the lncRNAs that were detected as expressed in PD blood leukocytes either pre- or post-DBS as well as in control (HC) leukocytes samples (Table S3B). Differential expression analysis of the PD brain-expressed lncRNAs (Fold change smear plot based on the tagwise dispersion analysis is given under Figure 3D) identified 242 lncRNAs as significantly altered in PD brains (FDR<0.05, Table S3C). Of these, 181 were up-regulated and only 61 were down- regulated in the PD brain samples (Figure S4 and Figure 3D). Of the overall 963 lncRNAs that were significantly altered in the PD brain RNA-Seq dataset (uncorrected p<0.05, Table S3D), 569 (59%) were also detected as expressed in the PD leukocyte RNA-Seq dataset. Of these, 135 have passed FDR threshold brain dataset (Table S3D). These included 2 of the lncRNAs that were significantly changed in PD patients leukocytes compared with HC leukocytes (FDR<0.05): RP13-507P19.2 and RP11-79P5.3, which were validated by qRT-PCR in both PD leukocytes and independent PD and unaffected control brain samples that were obtained from the Netherlands Brain Bank (NBB) (Figure 2D–2F). On the other hand, of the 13 lncRNAs that changed in PD leukocytes pre-DBS compared to HC, 7 were also detected overall as expressed in the independent PD brain RNA-Seq data set, 2 of which were also altered in the PD brain RNA-Seq dataset ((p<0.05, Table S3D and Table 1). We have validated one of these (RP11-79P5.3) by qRT-PCR in both the leukocytes and the independent PD brain sample set in both the amygdala and SN. Of all of the PD leukocyte-modified lncRNAs (having uncorrected significance level of p<0.05), 59 were significantly altered in the brain dataset as well (brain RNA-Seq data uncorrected p<0.05, Table S3D). Taken together, these findings highlight the relevance of the leukocyte-differentially expressed lncRNAs to the PD degenerative process overall.

### Characterization of secondary structures for two PD modified lncRNAs predicted to bind miRNAs

**Figure 4:**
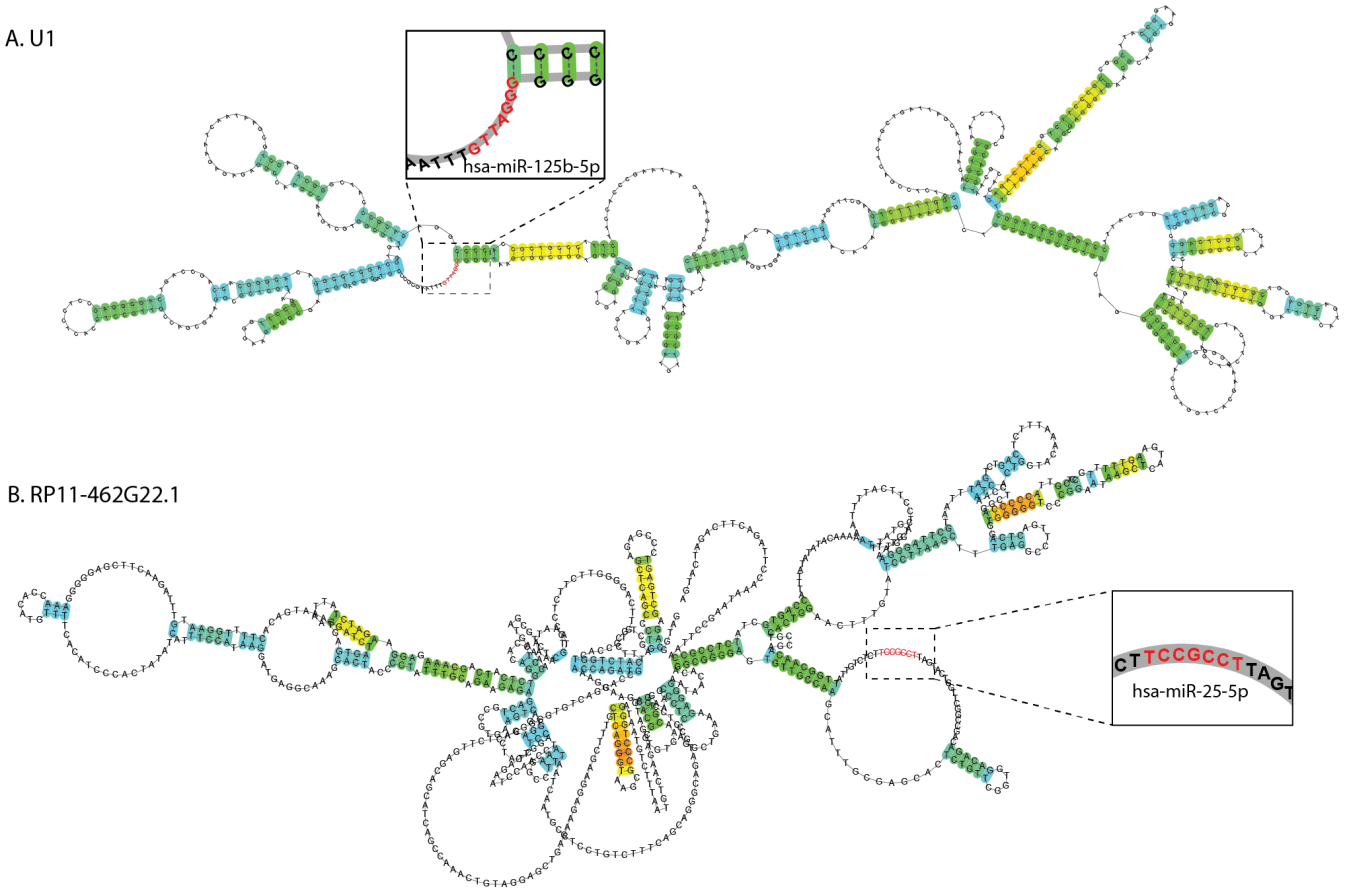
Secondary structures and miRNA-complementary regions of the PD- modified U1 and RP11-462G22.1 lncRNAs. The spliceosomal PD altered lncRNA U1 and the altered RP11-462G22.1 were predicted as targeting numerous microRNAs (miRNAs) by a method based on support vector machine that predicts both conserved and non-conserved miRNA targets through MirTarget2. The secondary structures of U1 (A) and of RP11-462G22.1 (lnc-FRG1-3) (B) were computed using RNAfold (minimum free energy: −412.07 and −271.93 Kcal/Mole, accordingly). Complementary seed-matching sites for one predicted miRNA were marked for each of the two lncRNAs: hsa-miR-125b-5p (A) and hsa-miR-25-5p (B), enlarged. Red nucleotide color marks the matched miRNA-seed lncRNA sequence region. In both cases, the predicted biding site was found in a single strand region predicted as having a loop structure. U1 was predicted as having a series of stem-loop structures, while RP11-462G22.1 exhibited a more complex structure compared to U1.

Since lncRNA functions are believed to be closely related to their secondary structures [66] [67], we applied a stochastic sampling method of structure prediction which maximizes the expected accuracy of the prediction [68]. The differentially expressed lncRNAs emerged as able to form complex stem-loop secondary structures. The secondary structure of U1 (also termed lnc-SPATA21-1) entailed a large number of stem-loop structures (Figure 4A) (predicted structure computed with [69], see methods for details). Two of the disease-altered lncRNAs, U1 and RP11-462G22.1 were predicted to target miRNAs and thus are potential competitive endogenous RNAs (ceRNAs). Using a support vector machine-learning algorithm (details under methods), U1 was predicted to bind 8 different miRNAs (listed under Table 1). These included hsa-miR-188-3p which controls dendritic plasticity and synaptic transmission [70], as well as hsa-miR-125b, which promotes neuronal differentiation and inflammation in human cells by repressing multiple targets [71] (Figure 4A, enlarged). RP11-462G22.1 (also termed lnc-FRG1-3) exhibited a yet more complex secondary stem-loop structure with a larger number of loops compared to U1 and was predicted to target 21 different miRNAs, identifying this lncRNA as well as a potential ceRNA (Table 1). These included the mitochondrial calcium import regulator hsa-miR-25-5p (complementary sequence enlarged under Figure 4B). Similarly to other lncrRNAs, which are generally poorly conserved between species, both U1 and RP11-462G22.1 were not found neither in the mouse nor in the zebra fish genomes [63].

### RNA-Seq analysis identifies known and novel junctions, exons and disease-related splicing events

Identifying the U1 spliceosomal lncRNA as disease- and treatment-altered in PD patients' leukocytes called for exploring possible concurrent splicing and transcript structure modifications in the same patients RNA samples. While we have previously reported alternative splicing alterations in PD leukocytes pre- and post-DBS through exon and junction microarray analyses [47], [48], RNA-Seq data analysis offers several important advantages in expression studies. Being independent of predefined probes (as compared with microarray platforms), RNA-Seq allows unbiased identification of novel junctions, exons and transcript isoforms as well as altered polyadenylation choices at high resolution. The in-depth coverage allows a more accurate measurement of exon inclusion or exclusion and identification of lowly abundant junctions and exons. Moreover, this analysis further enables detection of reciprocal pairs of junctions, one that includes and the other that excludes an exon. To fully exploit these virtues, we developed an updated version of AltAnalyze (version 2.0) for RNA-Sequencing analysis (http://www.altanalyze.org/). Extending the program that was initially developed and introduced for junction microarray analyses [37], [72], we introduce a significantly improved workflow (Figure 1). This is reflected in its user-friendly pipeline for the analysis of both known and novel splicing events directly from SOLiD BioScope processed RNA-Seq results (but also from other platforms, such as TopHat [73], HMMSplicer [74] and SpliceMap [75]). The obtained analysis results can be directly integrated with alternative exon or junction array datasets. Unlike existing RNA-Seq analytical pipelines, this new version of AltAnalyze can directly evaluate differential gene expression, identify known and novel alternative exons and junctions and alternative Poly-A sites, perform combinatorial exon and junction analyses and evaluate these effects at the level of protein domains, miRNA targeting and enriched biological pathways, as a fully automated and user-friendly pipeline.

We have developed a full analysis scheme to analyze using a wide range of analysis approaches gene, exon and junction levels of count data. This tool is able to analyze count information that is obtained from various platforms and analysis methods of data produced by RNA-Seq experiments (Methods). We have applied the full scheme of the new version of AltAnalyze (version 2.0) on leukocyte RNA-Seq data from PD patients pre- and post-DBS and matched HC (Figure 1). Splicing-Index (SI) algorithm (described in detail under [76] and [77]) was applied on the PD leukocyte RNA-Seq data based on the above-described novel targeted RNA-Seq module of AltAnalyze. The SI analysis enabled us to detect 1,652 alternatively spliced exons in 1,221 distinct genes (Table S4A and S4B) in PD as compared with HC, including such that belong to the splicing factor HNRNPF. The SI exon level analysis based on RNA-Seq read counts yielded many more splicing products in PD leukocytes' RNA than was previously detected by both our PD leukocyte microarrays (exon and junction) analyses [78]. Notably, 34 modified genes were detected in both the RNA-Seq and microarray technologies, including the elongation factor EIF2AK3 and the interleukin receptor IL1RL1. Also, the majority of disease-induced changes, 1313 were exon inclusion ones and only 339 – exclusion events, similar to the enrichment of inclusion events seen in cortical samples from Alzheimer's disease (AD) patients [79].

To further identify high confidence splicing events, we implemented linear regression analysis approach for analyzing all the reciprocal junction pairs detected in the RNA-Seq libraries (i.e reciprocal junction pairs: all the pairs of junctions one of which includes an exon in the transcript and one excludes it). Following sample-specific database construction of all the known and novel junctions available in the samples, the test is performed on all the junction pairs detected in the RNA-Seq libraries (both novel and known). Using this novel RNA-Seq implemented analysis approach on the sequenced PD leukocyte RNA, we could identify all the detected known and novel splice junctions. The novel RNA-Seq linear regression tool of the AltAnalyze RNA-Seq module subsequently identified 315 reciprocal junction pairs (Table S4C) as significantly changed in the disease, the majority of them (228 junction pairs) identified as novel by de-novo predictions that followed dataset-specific database construction (enabled in the new RNA-Seq adapted version 2.0 of AltAnalyze). Only 87 of the disease-altered junctions existed in current genomic databases, and thus the majority of these junctions could not be previously detected by microarray analyses. Of the total changed junctions, 157 and 158 spliced junction pairs induced exon inclusion and exclusion events in 144 different genes (Table S4D), suggesting that unique splicing events occur under PD. Some events were, however annotated as affecting more than one type of transcript structure variation, yielding an overall of 179 such PD-associated annotations. These primarily involved alternative cassette exons (N = 142, Figure S6). Other types of detected events included alternative 3′-ends (n = 11), alternative 5′-ends (n = 7), alternate promoters (n = 5), mutually exclusive exons (n = 2) and 2 trans-splicing inducing splice junction pairs events.

To enable a more rigorous detection of alternative exons, we implemented and applied for RNA-Seq the Analysis of Splicing by Isoform Reciprocit*y* (ASPIRE) approach [80] through the novel RNA-Seq version of AltAnalyze (version 2.0). This identified pairs of alternative exons and reciprocally expressed exclusion junctions through combined junctions and exons analysis based on RNA-Seq read counts for both types of gene structures. Subsequent analysis of the corresponding read counts from leukocyte RNA of PD patients compared to control samples identified 105 inclusion and 88 exclusion events in 192 exon-junction pairs (Table S4E).

### RNA-Seq analysis at both exon and junction levels identifies PD and DBS-induced splicing events

**Figure 5:**
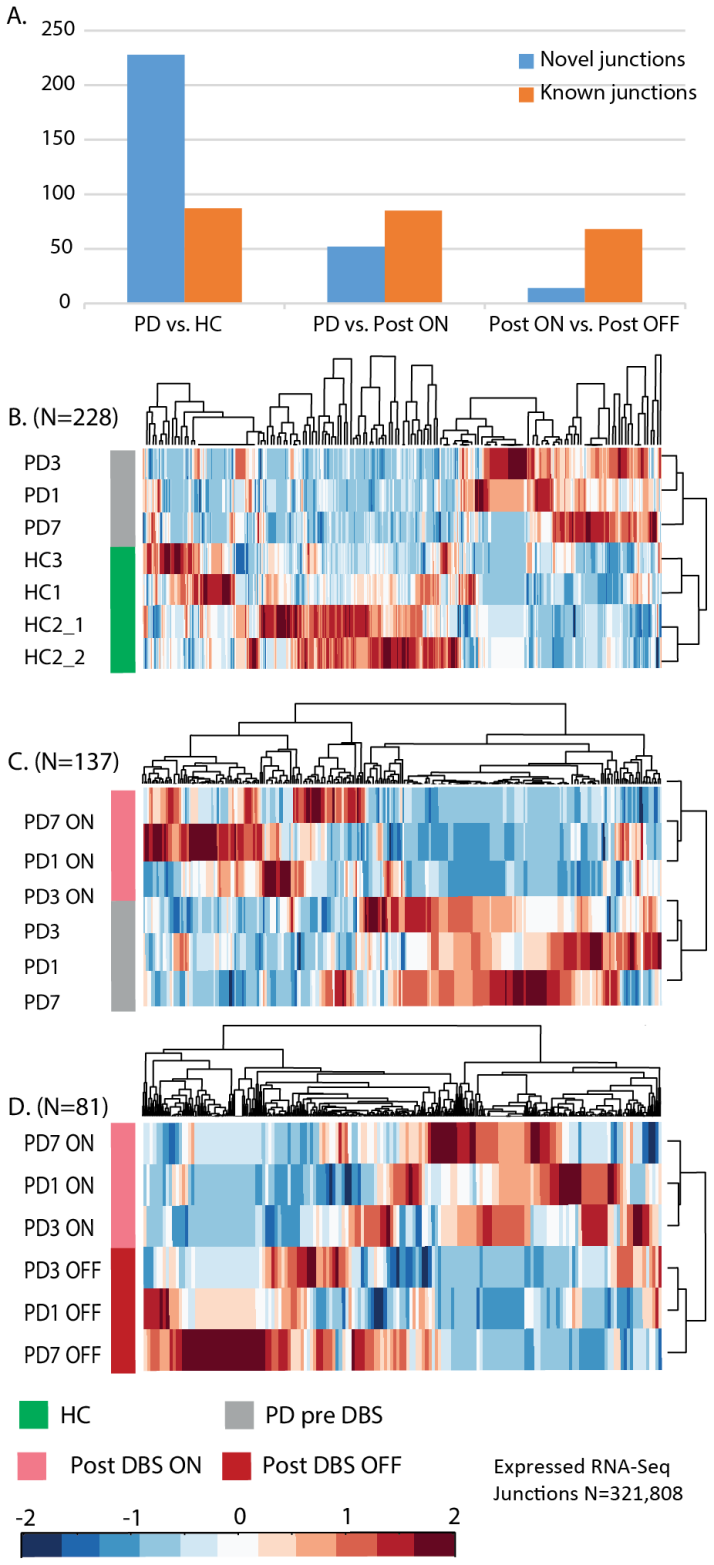
Alternatively spliced junctions detected in RNA-Seq libraries classify PD patients by clinical state. Among the junctions detected in each clinical stage, the disease exhibited more novel compared to known events (A, left bar). PD leukocytes pre-DBS exhibited more novel junctions as compared with PD patients' post-DBS (both on and off electrical brain stimulation). Hierarchical classification of alternatively spliced junctions in PD leukocytes that were detected by linear regression analysis distinguished pre-DBS patients from controls (B), post-DBS patients on electrical stimulation from pre-DBS ones (C), and post-DBS patients following one hour off electrical stimulation from the one hour earlier on stimulation state (D). The number of junctions detected as changed through linear regression analysis with the novel RNA-Seq module of AltAnalyze is given within parentheses.

Exon-level SI analysis of RNA-Seq libraries from RNA samples of patients pre-DBS compared to matched healthy control volunteers detected 1353 alternative events, in 1,069 distinct genes (Table S4F). Of these, 748 were inclusion and 320- exclusion events; extending our previous reports of widespread influence of DBS on splicing direction. Linear regression analysis showed DBS-induced massive reduction in spliced junction pairs, particularly the fraction of novel (compared to known) junctions in which splicing events were detected (Figure 5A). Hierarchical classification (HCL) of the PD and HC samples based on the expression of spliced junction pairs in the leukocyte RNA-Seq read counts, correctly classified patients from control samples (Figure 5B). The resolution of RNA-Seq is higher compared to microarrays, and the technology enables detection of novel gene structural elements and events. Nevertheless, changes in 20 of the RNA-Seq disease detected leukocyte genes were also detected as altered under PD in our previous exon array analysis of a larger cohort of PD patients pre- and post-DBS [47], [48]. These included the motor movement disorder dystonia related LRRC16A gene [81].

Linear regression junction level analysis of all the reciprocal junction pairs expressed in the RNA-seq data from the same patients, post-DBS treatment as compared with the RNA-Seq leukocyte data from the pre-DBS (disease) state revealed 137 pairs of AS changes (Table S4G). Of these, 85 were un-annotated in current genomic databases thus consisting of novel junction pairs (Figures 5A). The DBS alternatively spliced junctions were structurally a part of 73 different distinct genes (Table S4H). The detected events consisted of 53 exon-inclusion and 84 exon-exclusion events. The RNA-Seq read count of the DBS- altered reciprocal spliced junction pairs correctly classified patients post- from pre- surgical state (Figure 5C).

We implemented the robust ASPIRE [82] analysis approach for RNA-Seq data analysis and applied it on the RNA-seq data of patients' leukocytes post- compared to pre-DBS on stimulation while combining both exons and junctions count data for the analysis. This revealed 108 AS changes, 49 of those representing inclusion and 59 - exclusion events (Table S3I). These evented occurred in 17 genes that were also detected in our previous FIRMA analysis of exon microarrays data of a larger cohort of patients, including the inflammatory mediator IRAK1 [83]. Functional analysis using the AltAnalyze Gene Ontology (GO) [84] Elite module (GO-Elite) adopted for RNA-Seq detections highlighted enrichment in response to oxidative stress, ribonucleoprotein binding, transcriptional repression and histone methylation (Table S4O).

### A short one-hour electrical stimulation cessation modifies junction and exon splicing

Exon level analysis using SI measure of the RNA-Seq read counts revealed drastically reduced splicing changes following one hour of electrical stimulation cessation (OFF-Stim) compared to PD leukocytes from pre-DBS patients in 865 exons (Table S4J) of 778 genes (Table S4K). These included an inclusion event in the mitochondrial matrix gene Sirt3, which was recently found to induce aging-associated degeneration [85]. 496 (63%) of the off-stimulus detected alternative splicing events were exclusion events. Exon-level SI analysis detected changes in 11 genes that were previously detected by us in PD patient leukocytes through exon microarray analyses of a larger PD cohort (including the sequenced samples) [47], [48], including the ubiquitin-specific protease regulator USP13.

Linear regression analysis of all the reciprocal splice junction pairs found as expressed in the OFF- compared to ON-stimulus leukocyte mRNA-Seq data revealed 81 junction pairs as changed (Table S4l) in 36 genes (Table S4m); Of these, 68 were novel junctions, and 14 were known (Figure 5A, right bar graph). The altered junction pairs correctly classified leukocyte RNA from OFF-stim and ON-stim samples from one hour earlier (Figure 5D). A combined analysis of both exons and junctions quantified by the ASPIRE analysis yielded 70 OFF-stim induced junction level splicing changes, which included 28 inclusion and 42 exclusion events (Table S4N). Functional analysis through the GO-Elite module of AltAnalyze detected enrichment in immune effector process, natural killer cell proliferation, cytokine production, protein transport and regulation of response to stress (Table S4P).

### PD and DBS leukocyte splicing changes modify miRNA-targeted regions

Splicing modifications, and especially those affecting the 3′-untranslated region (3′-UTR) could potentially modify miRNA-binding sites. Implementing a module detecting miRNA enrichment in the novel version of RNA-Seq adopted program of AltAnalyze (details under http://www.altanalyze.org/), we identified potential miRNA binding sites in the regions detected through both junction- and exon-level splicing analyses (either through a single feature – exon/junction- or a dual feature, i.e pairs of junctions/exons). Enrichment analysis for potential miRNA binding sites in the genes detected as alternatively spliced by junction-level linear regression analysis of PD leukocyte RNA-Seq read counts compared with HC revealed 20 potential miRNA target sites in these genes (Table S5A). These spanned the two forms of hsa-miR-133, previously linked to PD (hsa-miR-133a, predicted to bind the GTPase GSN and the cancer-linked FRG1B [86], and hsa-miR-133b, also predicted to bind FRG1B. Enrichment analysis of miRNA binding sites in transcripts detected as alternatively spliced by exon level SI analysis of leukocytes from PD patients pre-DBS compared to controls detected 364 miRNA-target binding predictions (Table S5B), including the synaptic plasticity human hsa-miR-188 and the inflammation controlling hsa-mir-125. Enrichment analysis for miRNA-binding sites in the DBS-spliced genes detected by SI analysis predicted 481 such sites (Table S5C). These included predicted binding of spliced genes to three forms of hsa-miR-376 (a–c forms), hsa-mir-544 targeted at the same spliced gene (ITGAL) and the inflammation-related hsa-mir-150 [87] predicted to target the disease spliced gene FGD4. In comparison, the novel RNA-Seq linear regression AltAnalyze module identified only 5 miRNA-target pairs in DBS-modified transcripts (Table S5D). Also, the differentially spliced junction pairs detected by linear regression analysis showed no enrichment in miRNA binding sites when comparing post-DBS to one-hour stimulation cessation. Nevertheless, the splicing-index genes detected based on exon-level SI analysis of leukocyte read counts from patients post-DBS off stimulation compared to the on state exhibited 262 such predictions (Table S5E).

### PD and DBS splicing changes occur in predicted protein binding domains

Splicing modifications may further modify human protein binding domains. To assess the potential impact of the detected splicing changes on protein interactions, we tested for over representation of protein domains using z-score calculations (details under http://www.altanalyze.org/) for all the identified PD-related splicing events in the detected regions. The SI exon level analysis of PD compared to controls yielded 311 statistically significant (adjusted) changes in domain-target pairs (Table S6A), including metal binding domains and ubiquitin motifs. Similar analysis at the linear regression junction level of patients pre-DBS samples compared to HC yielded 16 statistically significant (adjusted) changes in domain-target pairs (Table S6B) (of a total of 12,529 domains analyzed), including metal-binding domains. Examples include the immune complement component ITGAX (also called CD11C) and ITGAM (also called CD11B). The DBS treatment induced changes in 351 domains in the transcripts detected by SI analysis of the RNA-Seq samples of patients post-DBS on stimulation compared to the pre-DBS state (Table S6C) and 11 domains in the transcripts detected by the linear regression junction level analysis (having adjusted p<0.05) (Table S6SD) out of a total of 12,539 protein domains detected in the RNA-Seq libraries. In contrast, the short one hour electrical stimulation cessation induced 281 predicted changes in functional binding domains (after p-value adjustment) in the linear regression detected junction pairs (Table S6E) and 44 enriched binding domains were detected by SI analysis on RNA-Seq detected exons that were modified upon electrical stimulation cessation post-DBS (Table S6F).

### PD isoforms are enriched in Prion-like protein domains and pathways

Mutations in prion-like domains of the splicing regulator heteronuclear ribonucleoprotein HNRNP A/B were recently linked with Amyotrophic lateral sclerosis (ALS) and rare proteinopathies [88]. Intriguingly, we detected PrLD domains that have RNA recognition motif (RRM) in 8 genes that were identified as undergoing alternative splicing modifications (in either exons, junctions or both) in PD patients leukocytes pre-DBS as compared with healthy control volunteers. Mutations in the Prion-like domains of three of these (FUS, EWSR1 and TAF15) (Supplementary Figure S5 and Table S7A) were recently reported as causally involved in other neuropathologies and human degenerative proteinopathies [50]. The Prion-like domains in PD-spliced transcripts included HNRNP A beta (HNRNP A/B) (Supplementary Figure S5). Correspondingly, analysis through the SI module for splice junction arrays by AltAnalyze of RNA samples produced from primary neurons of mice with ablated (KO) FUS or HNRNPA1 [89] detected 147 exclusion events (Table S7B). The identified genes included IMPA2, which is associated with schizophrenia and bipolar disorder [90], the enolase NO1 which is differentially expressed under stress [91], Mclf2, a rho guanine nucleotide exchange factor that interacts with the mental retardation and autism related gene interleukin-1 accessory protein-like 1 (Il1RAPL1), TMPR555, a trans-membrane serine protease whose presynaptic distribution on motor neurons in the spinal cord suggests an important role in neural development [92] and the JNK signaling pathway activator TCEA3 [93]. Additionally, splicing alterations of HNRNPA1 were previously associated with selective loss of HNRNP A/B and with massive exon inclusions in AD entorhinal cortex, and lentiviral-mediated suppression of HNRNP A/B impaired electrocorticography in the mouse brain [79].

ASPIRE analysis detected splicing changes spanning 151 transcripts in a splice junction microarray dataset of HNRNP A/B silencing of human embryonic kidney cells [94]. The affected genes included the nucleosome stability histone H2A, BCL2 which is involved in striatal neurons and considered to be a compensatory mechanism in PD [95], MLLT10 involved in lymphoblastic lymphoma [96], Chorod1 involved in brain development [97], the potential neuro-protector PON2 [98] and the iron homeostasis involved gene FBXL5 [99].

Of the genes detected as undergoing AS changes upon electrical stimulation cessation, 15 belonged to the proteins family that contains the RNA Binding motif RBM33 and included Prion-like domains or RNA Recognition Motifs (RRMs). These spanned RBM5, RBM19, RBM25 and RBM39, among others (Table S7A). In the RBM5, RBM19 and RBM25 genes, the prion-like domains were found as present both in altered exons as well as in exon-junction boundaries. Overall, six disease-spliced junctions were included in prion-like domain regions, including in the FUS and RBM33 genes, and 3 of the off-stimulus AS genes showed enrichment in prion-like protein domains (Prion-IPR000817).

### Disease-induced functional changes reveal PD and immune involvement

**Figure 6:**
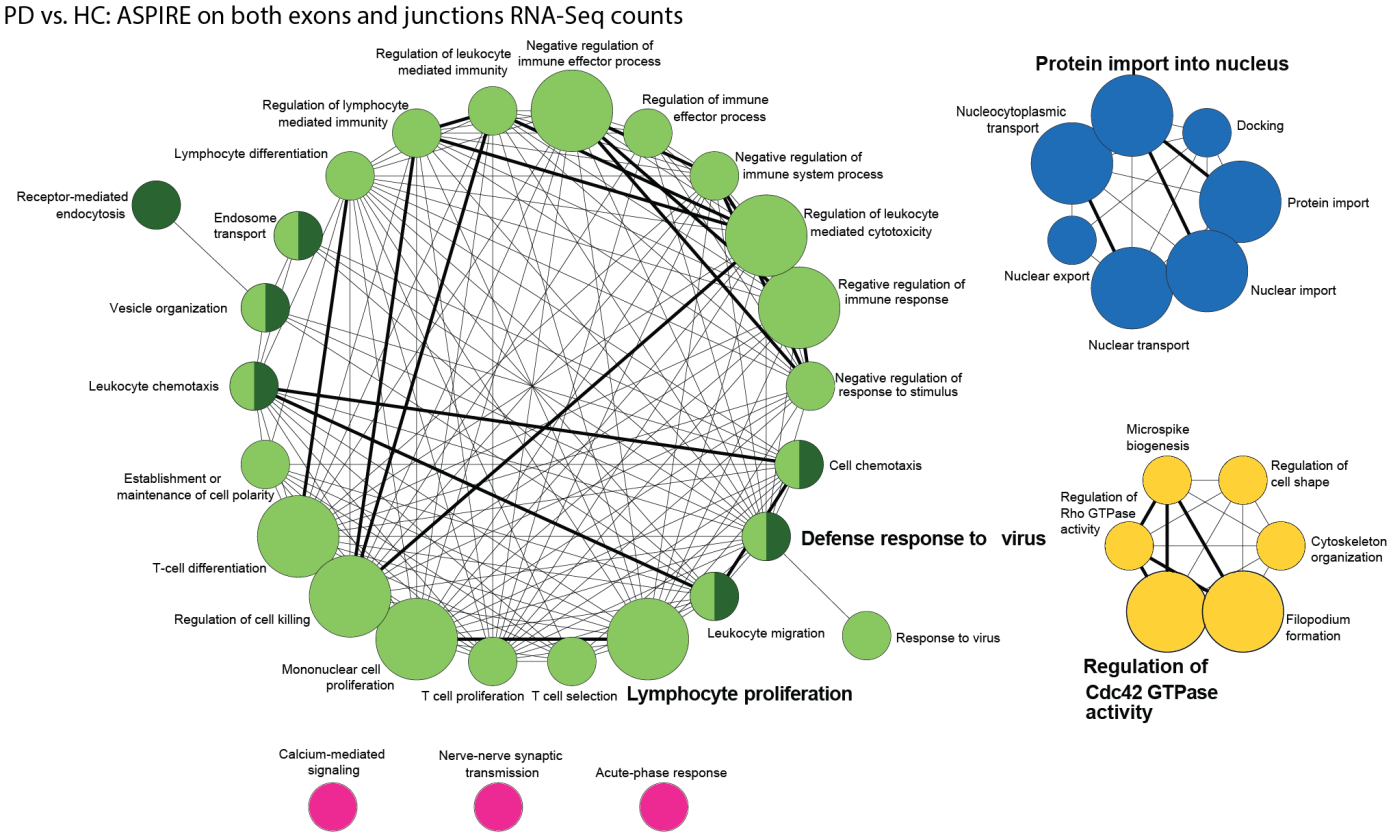
Network analysis identifies disease and treatment-relevant biological pathways. Network analysis of disease-relevant pathways identified disease involved processes. The network analysis was conducted on genes identified as undergoing splicing changes through ASPIRE analysis of both junctions and exons read counts. The analysis identified disease-modified biological processes included ATP processing, nuclear transport and immune-related events. The network was constructed using the ClueGO Cytoscape plug-in using KappaScore grouping, yielding 40 Gene Ontology (GO) terms functionally organized with 183 connections. Larger nodes (circles) indicate larger connectivity. Thicker edges marks higher statistical significance. The different functional groups are marked with different colors.

Functional enrichment analysis of the 114 disease-detected transcripts identified by the ASPIRE analysis on both exon and junction quantifications served to explore disease-related pathways. The analyzed transcripts were highly enriched in immune system pathways, including *regulation of leukocyte-mediated immunity* (Figure 6) as well as disease-related pathways such as *nuclear transport*, *regulation of GTPase activity* and *synaptic transmission*. Other affected pathways include protein import into the nucleus, known to be impaired in degenerative proteinopathies due to mutations in HNRNP A/B [50] as well as regulation of the neuro-immune CDC42 Rho GTPase; in the brain, CDC42 binds to collybistin and participates in bringing GABAergic receptors to anxiolytic synapses [100], whereas in lymphocytes it regulates cell division [101], perhaps explaining part of the immune mal-functioning that is a characteristic PD phenotype.

### Human leukocytes contain large cassette exons and other structural elements including alternative poly-A sites

**Figure 7:**
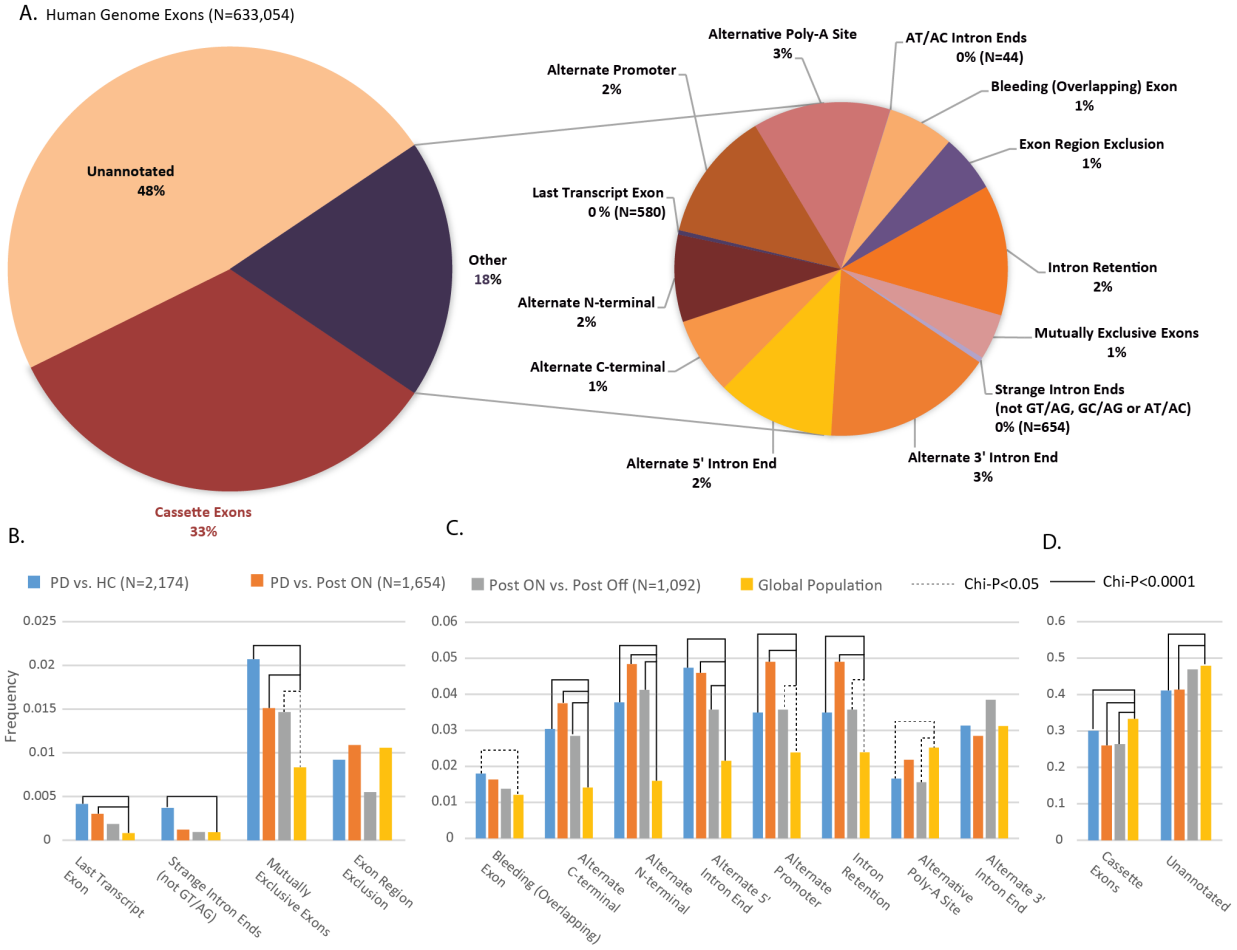
Differential promoter selection, splicing and polyadenylation choices in PD leukocytes pre- and post- DBS. (**A**) All of the exons expressed in all the sequenced libraries were annotated to transcript structure level. Cassette exons composed the majority of the exons (33%). 48% of the exons remained unannotated overall. The rest (18%) were sub-classified into different structural components (A, right pie, overall 13 types). These included intron retention (2%), alternative promoters (2%) and alternative polyadenylation (Poly-A) sites (3%). The frequency of distinct splicing events differed significantly in the disease (B–D, light blue bars), post-DBS on (orange) and off stimulation (grey) as compared with the global frequency of events (B–D, yellow bars). Shown are the rate events (B), the more common events (C) and cassette exons (D). Solid black line denotes goodness of fit significance level (solid: p<0.0001 and dashed 0.0001<p<0.05).

The disease effect at the transcript level was estimated based on structural elements for the global population of human genes. For this purpose, all the EnsEMBL (66) and UCSC (65) mRNA transcripts were compared to each aligned read identified in the RNA-seq samples. De-novo predictions for transcript level structure of all the human genome annotated genes using the constructed AltAnalyze database detected 633,054 known exons (of a total of 705,345 detected ones in the RNA-Seq samples). The detected exons were located in 3′ un-translated regions (3′-UTR), 5′-UTR, C- and N-termini, as well as in rare structural elements (such as nonconventional AT/AC ending introns). Overall, of 633,054 previously known and overall 705,345 exons detected in all the sequenced libraries, 343,400 exons (48%, Figure 7A, pie illustration on the left side) remained un-annotated at the level of transcript structure. The exons detected in all the RNA-Seq samples were primarily composed of cassette exons (33%, Figure 7A, left pie). The rest of the annotated exons (overall 18% of all the detected exons) were functionally annotated to 13 different transcript-level splicing structures (Figure 7A, right pie). These included alternate 3′ intron ends (3%), alternate 5′ intron ends (2%), alternate N terminals (2%), alternate C termini (1%), alternate promoters (2%), exon region exclusion (1%) and 580 last exons in the transcripts. 654 of the samples expressed exons detected in the RNA-Seq libraries were linked to strange intron ends (not GT/AG, GC/AG or AT/AC) and 44 to AT/AC intron ends. 1% of all the detected exons were bleeding exons (initiating or terminal exons that overlap with an intron of another transcript) and 1% were mutually exclusive expressed exons. Overall, 2% of the exons were linked to intron retention events and 3% - were located in alternative Polyadenylation (Poly-A) sites (Figure 7A).

### Alternative 5′ ends and 3′-polyadenylation variations in PD and DBS

Overall, the exons in samples of PD leukocytes showed significantly different frequencies of transcript functional relevance as compared with healthy samples (Figure 7B, light blue bars). A goodness of fit (Chi-square) test yielded a statistically significant difference between the distributions of specific events in the disease at the transcript level annotation (compared to the global population of exons detected in all the sequenced samples) for all the event types (except for alternate 3′ intron ends) (Figure 7B, black and dashed lines). The post-DBS patient leukocyte samples showed lower proportions of last transcript exons, strange intron ends, bleeding exons and cassette exons as compared with the pre-DBS (disease) state (Figure 7B, orange bars). In contrast, the proportion of exons annotated with other types of transcript level events (including alternate promoters and intron retention ones) increased following DBS (Figure 7B, orange bars). The short period of one hour of electrical stimulation cessation reduced the proportions of all the event types, as compared to the stimulated state (and in some cases, also compared to the pre-DBS disease state). However, the proportion of alternative 3′ intron ends was increased (Figure 7D and Table S8). The events with reduced proportions upon stimulation cessation included last transcript exons, strange intron ends, bleeding exons, alternate C and N termini, alternative 5′ intron ends and intron retention sites. The proportion of cassette exons remained similar in the off- compared to the on- stimulus state (but lower compared to the disease state in both).

Alternative Polyadenylation (Poly-A) predictions were incorporated into the novel version of AltAnalyze, which was adopted for RNA-Seq analysis and enabled reporting transcript event annotations. Exon regions overlapped with Poly-A binding sites that underwent alternative splicing modifications. A targeted analysis of the Poly-A sites in the sequenced libraries revealed increased alternative Poly-A site choices in PD leukocytes as compared with normal controls (Figure 7C, middle plot and Table S4). This increase was attenuated by the DBS treatment yet was largely regained (to even a higher proportion than in the disease) following one hour OFF stimulation (Figure 7C). It was previously shown that complex alternative RNA processing generates unexpected diversity of poly-A polymerase isoforms [102], which might be the case observed in the PD leukocytes RNA-Seq data.

## Discussion

We present here a comprehensive approach to analyze whole-transcriptome RNA-Seq data obtained via various platforms, using measurements of both splice junctions and exons, independently and in combination through various analysis methods, which enable identification and analysis of both know transcript variant as well as novel ones. The workflow enables additionally identification of transcript structures modifications, and integration with protein binding sites and microRNA annotations. We have re-implemented a diverse set of splicing-directed analysis methods (ASPIRE, linear regression, FIRMA and splicing-index) that were originally developed to analyze splice-sensitive microarray data [51] [103] [104], for the analysis of complex RNA-Seq data of protein-coding transcripts. The new RNA-Seq analysis workflow enables de-novo identification of genome-specific transcript structures through sample specific database construction based on the experimental specific read counts. We also incorporated for the first time prediction of Poly-A sites in the novel AltAnalyze version described here (version 2.0). This workflow enabled us to detect novel exons and junctions in protein coding RNA molecules, as well as a large range of splicing events under PD pre- and post- brain stimulation both on- and one hour off- electrical stimulation (which re-induces the disease motor symptoms), as compared with healthy control volunteers. We also present here a workflow for detection and differential expression analysis of lncRNAs in whole transcriptome RNA-Seq data.

Together, the novel analysis workflow and unique RNA-Seq dataset enabled us a widespread analysis of differential splicing as well as to detect lncRNAs and characterize their differential expression in both the disease and treatment states. At the transcript level, the DBS-induced increase in alternative ends, as well as in intron retention and alternative promoter usage, was accompanied by a 50% decrease in the number of ‘bleeding exons’ (that ‘leak’ into other transcripts). The number of cassette exons (present in certain transcripts but not in others) was predictably highest among all the possible types of splicing events in all the sequenced samples. Specifically, we observed increase in the frequency of cassette exons and intron retention events both in the disease and following DBS, as compared with the global population of expressed exons. Notably, non-conventional AT/AC and GT/AG ending introns were predictably very rare, in all the tested clinical conditions, as compared with the other types of transcript structural variations and disease-modified Poly-A choices.

Our deep survey characterized leukocyte-expressed lncRNAs in both patients and control volunteers and identified 5 lncRNAs that are over-expressed in the disease and inversely decrease following DBS. These include the spliceosome component U1, supporting the notion of disease-involved splicing modulations. Also, increased levels of the muscular dystrophy-associated RP11-462G22.1 (lnc-FRG1-3) may be relevant to the muscle rigidity in PD, one of the six disease hallmark motor symptoms. Another disease-modified lncRNAs that decreased post-DBS (RP11-79P5.3) was also found as differentially expressed by analysis of an additional external, independent PD brain RNA-Seq data-set [65] and its disease up-regulation was successfully validated by qRT-PCR in the leukocytes as well as in two brain regions from an additional set of PD and unaffected control brain samples, in both the Amygdala and SN.

So far, only a few large-scale studies have revealed fundamental characteristics of lncRNAs including their low levels of expression, temporal and spatial patterns of expression, sequence conservation and association with histone modifications [105]. Functional assays have also revealed diverse mechanisms through which lncRNAs act to regulate protein-coding genes at both the transcriptional and translational levels. However, to date there is insufficient data on the relationship between sequence, expression and pattern of newly identified lncRNAs [106]. The relatively low sequence and transcriptional conservation between species further complicate these studies. Yet, the identification of alternative, still unidentified features may produce a framework with which to accurately predict the functions of un-annotated lncRNAs [105]. An independent brain dataset analyzed in the current study exhibited a large number of lncRNAs commonly expressed in leukocytes from PD patients, thus we provide here an exceptionally rich resource for lncRNA expression in PD human leukocytes and brain regions.

We recently profiled differentially expressed miRNAs of PD patients' leukocytes pre- and post-DBS by small RNA deep sequencing [107], concurrently with alternative splicing changes of their predicted target genes. That study involved analysis by a junction array-adopted version of AltAnalyze. Here, we use the AltAnalyze target prediction module to detect potential miRNA binding sites within regions detected as undergoing splicing modifications by the RNA-Seq analyses, as well as putative protein binding domains. To detect disease and treatment-affected pathways, the splicing-sensitive results were re-analyzed using the functional AltAnalyze analysis module GO-Elite [108] for over-representation analysis (ORA) of pathways, ontologies and other gene sets. We believe that our current approach and results will provide a useful resource for biomedical researchers of movement and neurodegenerative disorders, and that our suggested analysis workflow may maximize the observations obtained by analyzing RNA-Seq data through simultaneous detection of novel junctions, exons and splice isoforms in a data-specific manner through comprehensive yet sensitive detection of alternative splicing events.

Our lncRNA analysis workflow and results will also provide an important resource to the biomedical community. Currently, 31% of the human genome bases in sequenced transcripts are annotated as intergenic (located between coding genes). Of these, lncRNAs are rapidly emerging as important and fascinating regulatory factors across a diverse catalogue of molecular, genetic and cellular processes, but phenotypic consequences of their differential expression, as well as sequence and structure derived functionality are still an Enigma. Here, in addition to comprehensive detection of both junction and exon level splicing changes in protein coding transcripts, we also fully characterized the disease- and treatment-expressed lncRNAs, and found large disease-induced expression changes in 13 lncRNAs (of the over 6000 lncRNAs detected in the leukocytes overall), including such that are involved in RNA processing. We have validated the RNA-Seq observed disease alternations through real-time RT-PCR for three lncRNAs, including two potential ceRNAs predicted to bind numerous miRNAs. Although only recently detected, lncRNAs raise a great interest to the scientific community due to their tremendous influence on our perception of genes. It is clear now that they can function at the molecular level [109], but their potential role in human neurodegenerative diseases was not reported yet. Certain lncRNAs function as transcriptional regulators of neighboring protein-coding genes by cis- or trans-modulation [110], enhance or repress nearby protein-coding genes [109], operate as epigenetic gene regulators through histone or DNA modification [111] (for example, in muscular dystrophy) [112], and act as precursors or decoys for small RNAs [113]. Thus, the expression map of lncRNAs in human leukocytes and specifically, in PD patients' pre- and post- DBS treatment may become an important resource. Specifically, both miRNA-binding lncRNAs and splicing modulations have been demonstrated to impact miRNA binding site integrity, which has been proposed to be an important mechanism in regulating miRNA-RNA sensitivity [51]. Emerging evidence further demonstrates a role for lncRNAs in regulating both miRNA targeting [114], possibly competing with the protein coding targets of the sponged miRNAs, and splicing factors [115]. For example, the lncRNA MALAT1 modulates SR splicing factor phosphorylation [116], whereas miR-188-5p which is complementary to the PD-induced lncRNAs targets the alternative splicing regulatory factor SFRS1 (SF2/ASF) [117] (which we previously reported as modified in PD patients through exon microarray analysis).

Two of the PD differentially induced lncRNAs predictably bind many complementary miRNAs, and were further increased following DBS treatment. RP5-875O13.1 (lnc-SPATA21-1) showed complementarity to 8 miRNAs and RP11-462G22.1 to 21 miRNAs, supporting the notion that lncRNAs may function in PD as protective decoys preventing the functioning of their complementary miRNAs. That miRNAs may present lncRNA-trapped, possibly non-functional versions, further suggests that quantifying miRNA levels in biological sources may be insufficient to predict their functioning potential. The DBS treatment potentially exacerbated this reaction, upon induction of changes in large number of lncRNAs. These two lncRNAs may hence belong to the newly discovered competitive endogenous RNAs (ceRNAs) lncRNA class, originally described as transcribed retropseudogenes that retain the miRNA-binding function of their parent mRNAs, which currently include lncRNAs [114]. CeRNAs have been proposed to function as miRNA ‘decoys’ or ‘sponges’, thereby de-repressing levels of protein coding transcripts that share with the ceRNAs the same miRNA response elements [114]. Although ceRNA-mediated regulation represents an elegant mechanism by which lncRNAs may control protein function through miRNA mediators, the proportion of lncRNAs that act as ceRNAs remains unknown [55].

Of note, secondary sequence structures were so far not studied for lncRNAs, and our current observation for secondary structure enabling possible miRNA sponging for two of the disease differentially expressed lncRNAs calls for future studies involving lncRNA secondary structures predictions. Future comparative study of various species will provide further insights into structure-based functionality of lncRNAs [118]. So far, Knockout models for specific lncRNAs did not produce any phenotypes. However, evidence for their importance stems from lncRNAs involvement in cancer and other human diseases, and evolutionary analyses suggest that lncRNAs represent a new class of non-coding genes whose importance should become clearer upon further experimental investigation [119]. We anticipate some of these associations will be made clearer by longitudinal studies that will include larger cohorts of PD patients as well as targeted lncRNA knockout models that will experimentally validate a link between splicing events with lncRNA differential expression. The discovery of at least one lncRNA regulated in our PD patients that affects splicing, highlights additional potential candidate lncRNA spliced targets consistently identified via RNA-Seq, junction and exon microarray analyses. Importantly, we found highly complex and previously unknown splicing and alternative poly-A patterns in healthy controls' leukocytes and a conspicuous decline of this rich variability in PD leukocytes. Together, these findings support the notion of a massive impact of both lncRNAs and the existence AS changes that cause a wide range of transcript-level structure modifications in PD.

Blood cells provide an accessible source for biomarker identification, and although accurate identification of disease biomarkers in the blood has proven difficult in the past, blood biomarkers were recently found for both neurological diseases as well as psychiatric disorders [34], [35], [36]. Future studies in larger cohorts of Parkinson's patients will enable verification of disease markers in the blood. Here, we employed a non-biased full leukocyte RNA-sequencing followed by detection of known and novel splicing events and transcript functional level annotations concurrently with detection of poly-A sites. This allowed us to profile both known and novel structural transcript changes in PD pre- and post-treatment ON- and OFF-stimulus at an unprecedented depth.

At the structural level, mutations in the prion-like domains of splicing factors such as heteronuclear ribonucleoprotein AB (hnRNP A/B) and FUS were recently shown to lead to pathological protein fibrils [50]. While their involvement in sporadic neurodegenerative processes is still incompletely understood, findings of hnRNP A/B decline in Alzheimer's disease [79] suggests that impaired splicing regulation might be involved in the emergence of sporadic neurodegenerative processes, including PD. Splicing alternations were also reported to occur early on in Alzheimer's disease (AD), and failed nuclear transport and fibril formation by splicing factors harboring prion-like domains, such as hnRNP A/B and FUS was recently implicated in Amyotrophic Lateral Sclerosis (ALS) [50]. It is hence noteworthy that we found AS changes in the prion-like domains of the non-mutated variants of these transcripts and identified *protein transport to nuclei as* a primarily impaired signaling network in PD leukocytes. Additional predictions involve neuro-immune signaling, with a specific focus on the CDC42 Rho GTPase which functions both in controlling anxiety and in defense against viral infection and general immune cell activities, both phenomena known in PD patients and which emerged in our network analysis as changed in the disease. Post-mortem brain studies of sporadic PD, highlighted mitochondrial dysfunction as being central to the disease [120], and it was further pointed out as contributing to the pathogenesis of other neurodegenerative diseases such as Ataxia [121]. Pink1 and Park2 may act in a quality control pathway preventing the accumulation of dysfunctional mitochondria, and regulators that control Park2 translocation into the damaged mitochondria were recently elucidated [120], revealing that this pathway is much more complicated than previously appreciated, and suggesting that other, yet unknown, regulators also contribute to the process.

Here, we have charted the first whole transcriptome genome-wide splicing map of Parkinson's leukocytes through characterization of both known and novel junctions and exons via multileveled analysis of high throughput long RNA sequencing. Wide annotations of alternate promoters, splicing and alternative poly-A sites allowed us to identify and quantify both disease- and treatment-induced splicing shifts, miRNA binding site modifications, putatively changed protein-protein interactions and other transcript structural changes in the three tested states of the participant patients (disease, post-treatment ON- and OFF- stimulus). We also noted shifts in splice patterns in PD leukocytes as compared with the global splicing map of the human genome, which was partially sustained post-DBS presenting specific attenuation of disease-derived increase in the frequency of Poly-A choices. To identify inclusion exons expressed in a reciprocal nature relative to a corresponding exclusion junction we have implemented and applied on the RNA-Seq data a more stringent algorithm based on the ASPIRE analysis approach. Importantly, we provide here a full resource of leukocyte-expressed lncRNAs in both disease and healthy states and specifically in PD.

In summary, we developed a novel computational approach and a user friendly tool for analyzing whole-transcriptome RNA-Seq data through sample specific database construction. Our workflow includes identification of novel splice junctions, exons and splicing events, including such that involve novel variants, in protein-coding genes. We combined both exon- and junction- level analyses by applying this newly developed version of AltAnalyze for RNASeq analysis to gain deep insight into gene expression and splicing aberrations in PD and search for electrical stimulation -induced changes, concurrently with global detection and differential expression of the leukocyte expressed lncRNAs. RNA-seq comprehensive analyses thus enable new insight to leukocyte transcriptome data, which becomes an important resource for researchers of neurodegenerative diseases overall, and our results will provide insights into DBS-treatable diseases overall (including mental disorders [122]). In particular, lncRNAs may be future novel biomarkers for PD and other neurodegenerative and neurological conditions and an important tool in future personalized neurology.

## Materials and Methods

### Ethics statement

PD patients and matched controls were recruited to the study according to the declaration of Helsinki (Hadassah University Hospital, Ein-Kerem, approval number 6-07.09.07) and have signed informed consent prior to inclusion in the study.

### Recruitment of human PD patients and control volunteers

Blood leukocytes were collected from 3 PD patients pre- and post- bilateral sub-thalamic (STN)-DBS neurosurgery while being on stimulation and following a short 1-hour of stimulation cessation and from 3 healthy age-matched control healthy volunteers (HC). The age, disease duration and Body-Mass Index (BMI) of the study patient participants are given under Table ST1. All the patient study volunteers that passed our stringent set of exclusion criteria signed informed consent forms prior to inclusion in the study (clinical parameters of the recruited volunteers are given under ST1). To control for variability in the leukocytes expression profiles that stem from other factors (such as infections, or other diseases), volunteers were assessed for their clinical background and state and fulfilled detailed medical history questionnaires. Exclusion criteria for participant patients included depression and past and current DSM Axis I and II psychological disorders (SM), chronic inflammatory disease, coagulation irregularities, previous malignancies or cardiac events, or any surgical procedure up to one year pre-DBS. Potential volunteers that did not fulfill these inclusion criteria were not recruited to the study. All patients went through bilateral STN-DBS electrode implantation (Medtronics, USA) and were under dopamine replacement therapy (DRT) both pre- and post-DBS (on significantly reduced dosage post-DBS with t-test p<0.01), the last medication administered at least five hours pre-sampling. The clinical severity of the disease was assessed by a neurologist by the Unified PD Rating Scale (UPDRS) [123]. Controls were recruited among Hadassah hospital staff and researchers at the Edmond J. Safra Campus (Jerusalem). All study volunteers underwent stringent filtering prior to inclusion in the study. The exclusion criteria for the healthy control volunteers included smoking, chronic inflammatory diseases, drug/alcohol usage, major depression, previous cardiac events, fever within up to three months prior to inclusion in the study and past year hospitalizations.

### Blood collection and leukocyte fractionation

Blood collection was conducted in a fixed range of hours (10AM–14PM). In order to reduce expression profile variability that depends on the time of sampling. To ensure accurate inspection of in-vivo leukocyte expressed RNA, the collected venous blood (9 ml blood using 4.5 ml EDTA (anti-coagulant) tubes) was immediately filtered using the LeukoLock fractionation and stabilization kit (Ambion, Applied Biosystems, Inc., Foster City, CA). To ensure high RNA quality, the leukocyte-enriched samples were immediately incubated in RNALater (Ambion) (http://www.affymetrix.com/support/technical/technotes/blood_technote.pdf). Stabilized filters and serum samples were stored at −80°C.

### RNA extraction from blood leukocytes

RNA extraction followed the manufacturers' alternative protocol instructions for RNA extraction from LeukoLock filters. Briefly, cells were flushed (TRI-Reagent Ambion) into 1-bromo-3-chloropropane-containing 15 ml tubes and centrifuged. 0.5 and 1.25 volume water and ethanol were added to the aqueous phase. Samples were filtered through spin cartridges, stored in pre-heated 150 µl EDTA; RNA was quantified in Bioanalyzer 2100. Determination of RNA quality and quantity were conducted using the Eukaryote Total RNANano 6000 kit (Agilent). RNA was frozen and stored in −80°C immediately after production.

### RNA-Seq libraries preparation and sequencing

RNA quality was assessed by running the samples on Agilent RNA 6000 Nano-gel (#5067-1511). For each library Ribosomal RNA of 5 ug total RNA was removed using Invitrogen RiboMinus kit (#A10837-08) and then sample was concentrated using the RiboMinus Concentration Module (Invitrogen). Ribosomal RNA removal was verified by RNA 6000 Nano gel analysis. Library construction was conducted according to SOLiD Whole Transcriptome Analysis Kit (PN4425680) protocol, fragmentation (by RNase-III) was verified on Agilent RNA 6000 Pico Kit (#5067-1513) and 150 ng fragmented RNA ware used for further protocols. cDNA samples were run on 4% Agarose gel, 150–250 base pairs (bp) sized fragments were cut and extracted using Qiagen Min-Elute Gel-Extraction Kit (#28604), gel was dissolved by intensive vortex and not by heating. Libraries were amplified for 12 cycles using bar-coded primers supplied in SOLiD Transcriptome Multiplexing Kit (Ambion, #4427046). Libraries were quantified using the Kapa ABI SOLiD Library Quantification Kit (KK4833) and diluted for final analysis on Agilent High Sensitivity DNA Kit (#5067-4626). 500 uM libraries were used for emulsion PCR according to Applied Biosystems SOLiD-3 System Template Bead Preparation Guide (4407421) to prepare for sequencing on the SOLiD-3 platform.

### Primary processing of SOLiD RNA-Seq reads

RNA-Seq reads (.csfasta files) and quality scores (.qual files) were obtained using the SOLiD instrument software: SOLiD-3. SOLiD-3. System software analysis was used for all the primary data analysis including image analysis, bead finding, quality metrics and color calls. The software applications used to set and control data analysis included SOLiD software suite under license agreement. The suit included: Instrument Control Software (ICS), SOLiD Experimental Tracking System (SETS), and SOLiD Analysis Tools (SAT) V3.0. Job management by the Job Manager used the Corona-Lite v4.0 platform. Sequencing was run on the Applied Biosystems SOLiD 3 System. Images of each cycle were analyzed, data was clustered and normalized. For each tag, a sequential (sequence-ordered) set of color space calls was produced. Quality metrics were produced through normalization. Two probe sets were used to maximize the fraction of “mappable” amplified beads, read length and sequencing throughput for sequencing of the 50-bp reads. Five rounds of primers (A, B, C, D and E) were used to sequence template by ligation of di-base labeled probes. As the libraries were size fragmented, the set of primers used was specific to the P1 Adaptor. For each library three types of raw data files were created: .csfasta (the sequenced reads in color space), .qual and .stats. The quality values given in the .qual files (estimate of confidence given for each color call), q for a particular call, is mathematically related to its probability of error (p), and is calculated as follows: q = −10log_10_p. The SOLiD q values are similar for those generated by Phred and the KB basecaller for capillary electrophoresis (described in detail under [124]). The algorithm relies on training (calibration) to a large set of control data and color calls for which the correct call is known. In SOLiD-3 system, the correct call is determined by mapping the read to a known reference sequence.

### Secondary RNA-Seq analysis: mapping of RNA-Seq reads to exons and junctions

The secondary data analysis included matching of the reads to reference genome and generation of base space sequences. Each library was mapped using SOLiD BioScope (v1.3) software (life technologies, applied biosystems, Carlsbad, California) via cloud computing. The count reads were mapped to the UCSC human genome version 19 (February 2009 GRCh37/hg19 assembly, homo_sapiens.GRCh37.56.dna.toplevel.fa database) twice: once to receive exon quantification (using the counttag tool) and once more to receive junction level quantification, using the BioScope splice junction extractor tool. The *.gff and *.sam files were created during this analysis step. The BioScope alignment software Mapreads was used. Count merging that employs discontinuous word pattern search algorithms was performed in both pipelines.

### Tertiary RNA-Seq analyses

#### 1. Predicting de- Novo junctions, exons and Poly-A site choices, annotating UCSC and EnsEMBL exons and detecting intron retention

For each RNA-Seq sample, a sample-specific database was constructed, based on the count reads obtained by the experimental procedure, and both EnsEMBL and UCSC genome coordinates. Following the identification of all of the transcript structural event types by parsing of all EnsEMBL and UCSC databases, the cases of intron retention were identified first by searching for regions that consist of a single exon and span two adjacent exons in at least one additional transcript for each gene. The remaining exons were clustered based on genomic coordinate overlaps (e.g alternate 5′ or 5′ start-sites). Each exon was annotated as corresponding to exon block and region number within the corresponding transcript. All possible pair-wise transcript comparisons for each gene were then performed to identify exon pairs that show evidence of alternative exon-cassettes, alternative 3′ or 5′ splice-sites or alternative-N or -C terminal exons. All transcript exon pairs were considered (except for those adjacent to a retained intron) by comparing the exon block-ID and region-IDs of an exon and its neighboring exons to the exon blocks and regions in the compared transcripts, and a custom heuristic assigned the appropriate annotation based on these transcript comparisons. In addition to all of the de-novo splicing annotations, additional splicing annotations were imported from the UCSC genome database and linked to existing exon blocks and regions based on genomic coordinate overlaps. Various programs can be used to produce the junction input .BED/TAB files, in addition to SOLiD Bioscope output files (such as TopHat [73] (http://tophat.cbcb.umd.edu, HMMSplicer [74] (http://derisilab14.ucsf.edu/software/hmmsplicer)). Paired-end data from SpliceMap [125]) can also be analyzed. Exon count input .bed files are produced from BAM files with BEDTools (and exon counts can be further uploaded as .tab count files, produced by BioScope for SOLiD libraries, as in this case). In addition to known splice junctions (EnsEMBL and UCSC), novel reciprocal junction and trans-splicing events are analyzed and annotated as well. We have further incorporated, for the first time, alternative Poly-A predictions using the polyA database [126] with human genomics coordinates. Additionally, alternative promoters were identified. Further details are given under AltAnalyze online documentation (www.altanalyze.org).

#### 2. A novel AltAnalyze RNA-Seq module enables de-novo detection of junctions, exons and alternative splicing events and prediction of alternate Poly-A sites, promoters and intron retention events

To identify alternative splicing events, we have adopted for RNA-Seq analysis the AltAnalyze program (originally developed for Affymetrix mouse custom splicing arrays [72] and further on to prototype Affymetrix human splice junction arrays [37]). For the analysis of RNA-Seq data, we implemented both single-feature (exon/junction) through Splicing-Index (SI) (initially introduced to exon microarrays [77]) and FIRMA, or combined (pairs) of features analyses (e.g reciprocal junction pairs): through linear regression and ASPIRE. The calculation of statistical significance involved t-test (default) but can also be modified to other statistical tests (such as Kolmogorov-Smirnov). In addition to detection of known junctions and exons, novel junctions and exons (as well as splicing changes) are derived by de-novo predictions. The exon values are normalized to the total number of read counts using the RPKM calculation prior to the analysis. Further details are given under AltAnalyze online documentation (www.altanalyze.org). The tool also enables concurrent analysis of junctions and exons – combined gene-level analysis, and permutations for junction-level analyses. The FIRMA approach [104] was implemented for gene-level analysis of RNA-Seq data. We applied both junction and exon level analysis approaches on this study RNA-Seq data from PD leukocytes, to conduct three independent comparisons: patients pre-DBS to healthy control volunteers, patients post-DBS on stimulation to their pre-DBS state, and post-DBS following one hour off electrical stimulation as compared with one hour earlier while being on electrical stimulation. Predictions of alternate Poly-A sites in the sequenced samples were enabled by incorporating polyA-database analysis (http://polyA.umdnj.edu).

#### 3. Analysis of miRNA and protein binding domain composition in spliced regions

Enrichment of both protein domains and miRNA binding sites in transcript regions that were detected as alternatively spliced was conducted through the novel targeted RNA-Seq AltAnalyze V2.0 module. To identify alternative protein domains, RNA-seq and microarray probe-set sequences were used to identify which protein domains align to, or are missing from, spliced transcripts for each disease, treatment or stimulation cessation samples, and specifically for each spliced isoform. Further details are given under AltAnalyze online documentation (www.altanalyze.org).

#### 4. Frequency and goodness of fit analysis of alternative splicing and other transcript structure modifications

We compared the frequency of each type of exon level splicing event in PD patients pre-DBS compared to healthy control volunteers, patients post-DBS on stimulation compared to pre-DBS and post-DBS following a one hour of stimulation cessation compared to post-DBS on stimulation, using the standard chi square goodness of fit test.

#### 5. Functional GO analysis

Gene Ontology (GO)-Elite pathway analysis was conducted through the AltAnalyze program on the analyzed output result files of the detected splice events at both the junction and exon levels. Additionally, functional enrichment was assessed through the EASE [127] functional classification tool. The network analysis was conducted using the ClueGo [128] plugin of Cytopscape [129] using kappa score and the GO database.

### Detecting long non-coding RNAs (lncRNAs) in the RNA-Seq libraries

The bed coordinates of the Gencode v. 7 human long non-coding RNAs database were downloaded from the GENCODE lncRNA data page of the CRG Bioinformatics and Genomics Group [http://big.crg.cat/bioinformatics_and_genomics/lncrna_data] and complemented with other non-coding transcript information available from the EnsEMBL BioMart version 0.7 query interface to the EnsEMBL Genes 72 – GRCh37.p11 database (www.ensembl.org) Genome coordinates in bed format corresponding to the mapped reads for all samples used the Lifescope Lifetech 2.5.1 software and UCSC hg19 masked reference database as obtained by the original .sam files with SAMtools, SAMtools view and bedtools bamToBed. These read bed files were intersected with the genome coordinates of the above-mentioned lncRNAs using the bedtools intersectBed program, requiring a 90% overlap of each sequence read with a target lncRNAs. Lists of sequence tags corresponding to lncRNAs were obtained by intersection of the bed tools.

### Differential expression analysis of leukocyte LncRNA count reads

The count information of all of the detected leukocyte lncRNAs was first filtered. LncRNAs that did not present read count in three or more libraries, and ones that did not exist in EnsEMBL were filtered out. The remaining lncRNAs (overall, 6430) were analyzed using the Bioconductor edge-R [59] software version 3.0.1 to detect differential expression in PD patients pre-DBS compared to healthy control volunteers, post-DBS on stimulation as compared with pre-DBS state and post-DBS off electrical stimulation as compared with post-DBS on electrical stimulation. This analysis module is particularly suitable to use on small number of rate replicate samples. The results were annotated using the BioMart integrated annotation database query interface [130], using the human genome reference consortium assembly build version 37 (GRch37, hg19) and GENCODE version 7 [131].

### Secondary structure prediction of LncRNAs and lncRNA miRNA binding prediction

A method that maximizes the pseudo-expected accuracy of the model served for prediction of RNA secondary structure [68]. The binding affinity of the lncRNAs to potential targets as sponge was computed using MirTarget2 [132], which implements a support-vector-machine (SVM)-based miRNA target prediction algorithm that scans all of the seed-matching sites in the potential targets and predicts both conserved and non-conserved miRNA targets in mammals.

### Human brain samples

Dissected brain tissues (amygdala and SN) from PD patients (n = 5, 4 males and 1 female) and unaffected (non-demented) controls (n = 5, 3 females and 2 males) were provided by the Netherlands Brain Bank (NBB) (ST9). Ethical approval and written informed consent from the donors or the next of kin was obtained in all cases. These tissues were kept at −70 degrees until use and served for RNA extraction and qRT-PCR validation tests.

### RNA extraction form human brain samples

RNA was extracted from brain tissues using the QIAGEN (Venlo, Netherlands) easy kit, which ensures full representation of all RNA length groups. Briefly, brain tissue was homogenized with 700 µL QIAzol lysis buffer and subsequently lysed for 5 minutes and mixed with 140 µL of chloroform to allow full neutralization. Centrifugation for 15 minutes (in 12,000× g and 4°C) followed suspension of 3 minutes. The aqueous phase was mixed with 1.5 volume of ethanol, loaded on RNA-binding spin column and centrifuged for 30 seconds in 8400× g. The column was washed with 700 µL RWT buffer (85% ethanol) and twice with 500 µL RPE buffer (QIAGEN, 70%), for the removal of DNA and protein remnants, respectively. Washing was performed by centrifugation for 30 seconds in 8400× g and discarding the flow-through. Finally, spin column was centrifuged for 1 minute in 21,067× g to further dry the column. 50 µL of nuclease-free water were used to elute the RNA, which was immediately put on ice to prevent degradation. The RNA concentration was determined by Nanodrop-1000, and its integrity was assessed with 1% agarose gel and identification of two distinct bands (28S and 18S rRNA).

### cDNA synthesis from brain and leukocyte human RNA samples

The DNA remnants were degraded using Sigma Aldrich (3,050 Spruce St., St. Louis, Missouri 63,103, United States) DNAse1 Amplification Grade (Sigma Aldrich). 800 ng of RNA were diluted in 8 µL of nuclease-free water, and then mixed with 1 µL of DNAse and 1 µL of 10× reaction buffer (Sigma Aldrich (3,050 Spruce St., St. Louis, Missouri 63,103, United States)). Following 15 minutes of incubation in 25°c, 1 µL of stop solution (Sigma Aldrich (3,050 Spruce St., St. Louis, Missouri 63,103, United States)) was added, and the mixture was incubated for 10 minutes in 70°c on a MJ Research PTC 200 Thermal Cycler (GMI Inc., 6511 Bunker Lake Blvd, Ramsey, MN 55303, United States). The entire volume of the mixture (800 ng of RNA in 11 µL volume) was used for cDNA preparation using the Quanta qScript cDNA Synthesis Kit (Quanta biosciences Inc., 202 Perry Parkway, Suite 1. Gaithersburg, MD 20877, USA). For each reaction 11 µL RNA were mixed with 4 µL ×5 Reaction Buffer, 4 µL Nuclease-Free Water and 1 µL Reverse Transcriptase (except for the no-RT controls, where reverse transcriptase was not added), for a final 20 µL reaction volume. Mixture was put in a 200 µL PCR tube, and placed in a MJ Research PTC 200 Thermal Cycler (GMI Inc., 6511 Bunker Lake Blvd, Ramsey, MN 55303, United States) programmed for 5 minutes in 22°c, 30 minutes in 42°c and 5 minutes in 85°c. cDNA was then diluted 1∶10, by adding 180 µL DDW.

### Quantitative real-time PCR and primers

iTaq Universal SYBR green supermix 2× (Biorad Inc., Hercules, California, US) was used for both the target and reference genes used for normalization. 10 µL of SYBR supermix (Biorad Inc., Hercules, California, US), 1 µL of 10 µM of each left and right primers, and 8 µL of cDNA were used for each reaction. Reaction was performed on a Biorad (Hercules, California, US) CFX96 Touch Real-Time PCR Detection System. The protocol used for product amplification was 95°c for 3 minutes, 95°c for 15 seconds and 51 repeats of 60°c for 30 seconds, then melting curve was performed by increasing the temperature from 67.0 to 94.6°c in 0.3°c increments every 5 seconds. The data was obtained using Bio-Rad CFX Manager 3.0 software. For each primer pair and tested samples, triplicate PCR reactions were tested. Triplicates that were not tightly grouped (more than 1.5% cycles apart) were removed from further calculations as outliers. If the triplicates were not tightly grouped, and no outlier could be identified, the triplicate was re-run (and the whole sample was omitted from further analysis if the outcome of the re-run still showed un-tight grouping). The primers (Sigma Aldrich (3,050 Spruce St., St. Louis, Missuri 63,103, United States)) used for the lncRNA targets and reference genes are as follows: RP11-462G22.1 – forward primer GAGCTGCCTTTCATCTGGTC, reverse primer GGTAGTGCTTTGCCTCATCC; U1 – forward primer GAACCCCGAGTCCACTGTAA, reverse primer TGAACCCCGTTATGTCAGGT; RP11-79P5.8 – forward primer CTCGGCTTCGACTTTAGCTG, reverse primer CTTCTTTTTCACCGCTCCTG; TUBB3 – forward primer GCAACTACGTGGGCGACT, reverse primer GGCCTGAAGAGATGTCCAAA; RPL-19 – forward primer GCTCGATGCCGGAAAAACAC, reverse primer GCTGTACCCTTCCGCTTACC.

### Genome annotation of lncRNAs in both PD leukocytes and brain RNA-Seq dataset

The sequences (in fastq or Color Space format) were mapped against the GENCODE V7.0 reference database using the Bowtie aligner software [133] version 1.0.0 against the FastA files from GENCODE lncRNA catalogue at CRG (http://big.crg.cat/computational_biology_of_rna_processing/lncrna). This target dataset consisted of 14,880 sequences in FastA format and was used also for the leukocyte RNA-Seq data alignment. The run parameters were set as in the alignment of the leukocyte RNA-Seq count reads (-k1 and –best). The aligned sequences were parsed with in-house generated scripts (Genomnia srl, Milan, Italy) and transformed in transcript count tables with EnsEMBL GENCODE transcript ID identifiers. Several transcripts that were not included in EnsEMBL were eliminated from the dataset prior to the analysis (since considered as ‘retired’ transcripts). Cross datasets comparisons were performed with the VLOOKUP Excel function based on the ID column.

### Differential expression analysis of lncRNAs in PD leukocytes and brain samples

Differential expression analysis of PD samples compared to normal controls was performed with the Bioconductor EdgeR software [59] version 3.4.2 on R (version 3.0.2, 64 bit), using Trimmed Mean of Ms (TMM) normalization [60] and exact test. The tagwise dispersion was calculated for each lncRNA (Supplementary Figure S3, PD leukocyte compared to controls as an example), and was moderated by EdgeR toward a common value inferred by all the examined genes. The dispersion parameter determined how to model the variance for each gene in each comparison and dataset. The common variation is inferred from all the datasets. The variance under a negative binomial model computed as 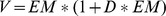 where EM is the estimated mean and D – the dispersion, for each gene in each comparison and dataset. The fold change calculation uses the square root of the dispersion as the biological coefficient of variation, inferred by Poisson distribution), and the tagwise dispersion parameter determines how to model the variance for each gene for the differential expression analysis.

### Accession numbers

All the raw and processed RNA-Seq whole transcriptome profiling files (.csfasta, .qual, .stats, .gff, .bam, .tab, .contig.range and .bed files) were deposited under the Gene Expression Omnibus depository (GEO) [52] and are available under series accession number GSE42608. The independent PD brain RNA-Seq dataset was obtained from the Array Express repository [134] (accession number E-GEOD-40710).

## Supporting Information

Figure S1 **Biological Coefficient Variation (BCV) and tagwise dispersion plots for PD pre-DBS and HC leukocyte lncRNAs analyzed from RNA-Seq count reads.** (A) The biological coefficient of variation (BCV) plot is given for the RNA-Seq samples from blood leukocytes of PD patients and age- and gender-matched healthy control (HC) volunteers is given. X axis: log count per millions (CPM). Y axis: the biological coefficient of variation (BCV) - the statistic of the dispersion (defined as the ratio between the standard deviation and the mean). (B) Multidimensional scaling (MDS) clustering plot of lncRNAs detected as having a leading fold change (calculated by EdgeR in RNA-Seq samples from leukocyte RNA-Seq libraries of PD patients' pre-DBS and HC). The location represent the similarity between the corresponding samples. X axis (leading fold change dimension 1) is the direction that best separated the samples, regardless of type. Y axis (dimension 2) is the best direction, uncorrelated with the first, which separates the samples.
(TIF)

Figure S2 **Biological Coefficient Variation (BCV) and tagwise dispersion plots for lncRNAs detected in PD brain RNA-Seq data compared to HC brain samples.** (A) The BCV plot is given for the RNA-Seq samples from brain samples of PD patients and unaffected (HC) individuals (accession number E-GEOD-40710). X axis: log CPM. Y axis: the biological coefficient of variation. (B) MDS plot leading fold change of lncRNAs detected as modified (by EdgeR analysis) between PD patients pre- and post-DBS in RNA-Seq libraries from blood leukocytes RNA. The location represent the similarity between the corresponding samples. X axis: dimension 1, y axis: dimension 2.
(TIF)

Figure S3 **Biological Coefficient Variation (BCV) and tagwise dispersion plots for PD pre- compared to post-DBS leukocyte state of lncRNAs analyzed from RNA-Seq count reads.** (A) The BCV plot is given for the RNA-Seq samples from blood leukocytes of PD patients pre- DBS and post-DBS on electrical stimulation. X axis: log CPM. Y axis: the biological coefficient of variation. (B) MDS plot leading fold change of lncRNAs detected as modified (by EdgeR analysis) between PD patients pre- and post-DBS in RNA-Seq libraries from blood leukocytes RNA. The location represent the similarity between the corresponding samples. X axis: dimension 1, y axis: dimension 2.
(TIF)

Figure S4 **Biological Coefficient Variation (BCV) and tagwise dispersion plots for PD patients post-DBS on stimulation compared to following one hour of electrical stimulation cessation (OFF-state).** (A) The BCV plot is given for the RNA-Seq samples from blood leukocytes of PD patients post-DBS on and following one hour of electrical stimulation cessation. X axis: log CPM. Y axis: the biological coefficient of variation. (B) MDS plot leading fold change of lncRNAs detected as modified (by EdgeR analysis) between PD patients post on-stimulation and following one hour off- electrical stimulation of RNA-Seq libraries from blood leukocytes RNA. The location represent the similarity between the corresponding samples. X axis: dimension 1, y axis: dimension 2.
(TIF)

Figure S5 **Alternatively spliced neuropathologies-related genes which include prion-like domains.** Prion-like protein domains were found as enriched in previously neuropathology-related genes, including FUS, EWSR1, TAF15 and the HNRNP A/B. Gene structures include white boxes for untranslated regions, black boxes for translated exons and lines for introns. The junction probe-sets that were identified as differentially expressed are marked in solid (p<0.05) or dashed (p<0.001) lines.
(TIF)

Figure S6 **PD splicing modulated transcript regions.** The distribution of different splicing event types detected in PD patients' blood leukocytes as compared with matched healthy control volunteers through linear regression analysis of all the reciprocal junction pairs expressed in the corresponding RNA-Seq libraries included cassette exons (the majority), alternative terminals, alternative promoters and alternative polyA- site usage.
(TIF)

Table S1 **RNA-seq libraries read counts and genome mapping statistics, and clinical parameters of the patients and control volunteers from which blood leukocyte RNA was collected.**
(XLSX)

Table S2 **RNA-Seq analysis results of PD patients' blood leukocytes detects lncRNAs (pre- and post-DBS on and off stimulation).**
(XLSX)

Table S3 **Detection and differential expression analysis of lncRNAs in PD brain RNA-Seq data.**
(XLSX)

Table S4 **Alternative splicing events in PD leukocytes pre- DBS compared to HC, post-DBS on stimulation compared to pre-DBS and following one hour off electrical stimulation compared to on stimulation, were detected through a variety of analysis approaches including Splicing Index, Linear Regression, FIRMA and ASPIRE on both junction and exon levels as well as combined. Functional GO analysis results are given as well.**
(XLSX)

Table S5 **Enriched miRNA binding sites within alternatively spliced genes in PD detected splicing events.**
(XLSX)

Table S6 **Protein domains found as enriched in PD alternatively spliced genes altered pre- and post-DBS on and off stimulation, and alternatively spliced genes detected by exon array analysis following knock down of prion-like domain containing proteins involved in neuropathies.**
(XLSX)

Table S7 **PD leukocyte alternatively spliced genes contain Prion-like domains.**
(XLSX)

Table S8 **Various types of splicing events in PD leukocytes pre- and post-DBS detected by RNA-Seq analysis.**
(XLSX)

Table S9 **Netherlands Brain Bank (NBB) PD brain samples clinical details.**
(XLSX)
