# Correction: Long Non-Coding RNA and Alternative Splicing Modulations in Parkinson’s Leukocytes Identified by RNA Sequencing

**DOI:** 10.1371/journal.pcbi.1003647

**Published:** 2014-05-16

**Authors:** The PLOS Computational Biology Staff

The images for Figures 2 and 3 are incorrectly switched. The image that appears as Figure 2 should be Figure 3, and the image that appears as Figure 3 should be Figure 2. The figure legends appear in the correct order.
