# Supplementary figures and images for: Long Non-Coding RNA and Alternative Splicing Modulations in Parkinson's Leukocytes Identified by RNA Sequencing
Source: PLoS Comput Biol. 2014 Mar 20;10(3):e1003517. doi: 10.1371/journal.pcbi.1003517 (PMC3961179; doi:10.1371/journal.pcbi.1003517)

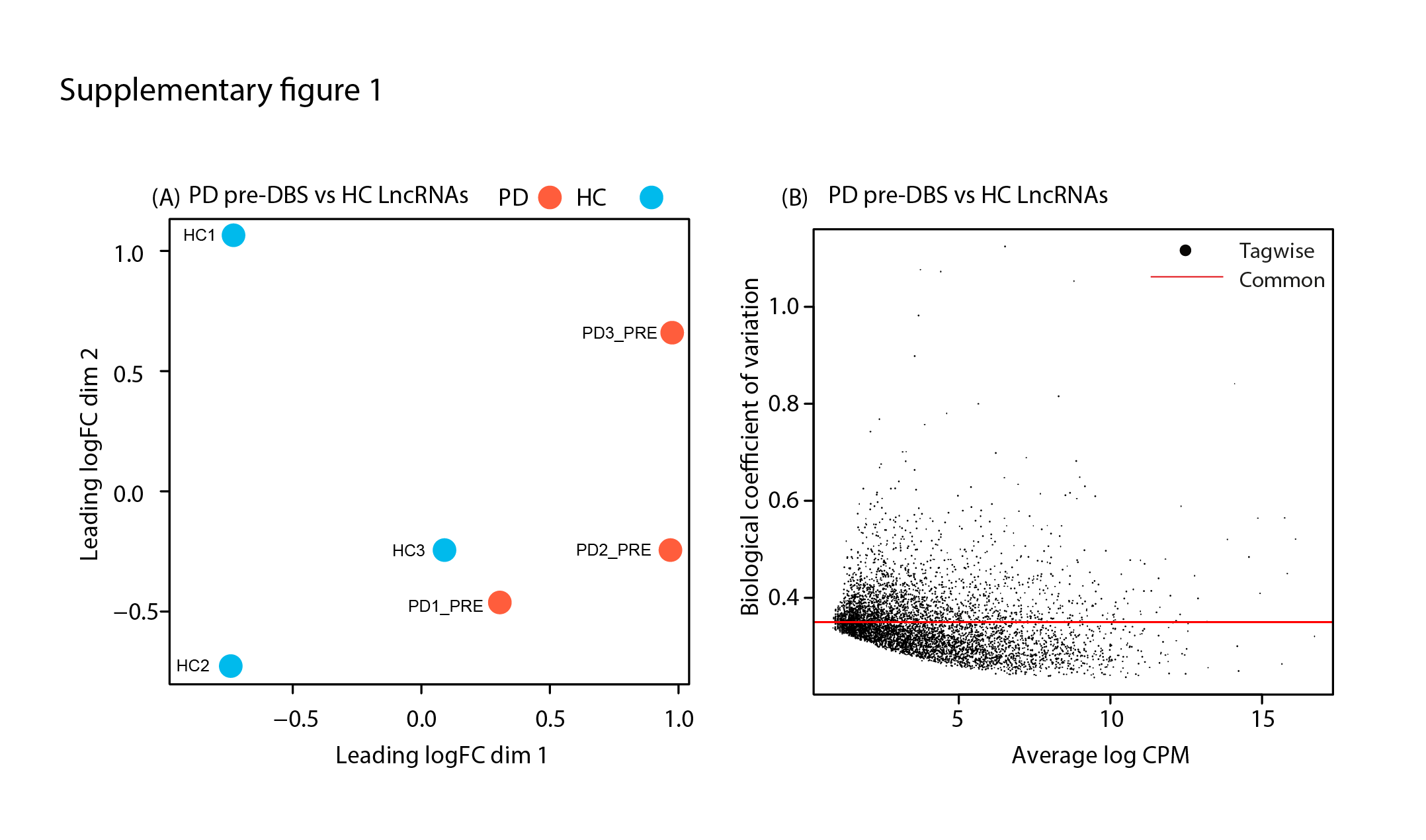

Supplement: Figure S1 — Biological Coefficient Variation (BCV) and tagwise dispersion plots for PD pre-DBS and HC leukocyte lncRNAs analyzed from RNA-Seq count reads. (A) The biological coefficient of variation (BCV) plot is given for the RNA-Seq samples from blood leukocytes of PD patients and age- and gender-matched healthy control (HC) volunteers is given. X axis: log count per millions (CPM). Y axis: the biological coefficient of variation (BCV) - the statistic of the dispersion (defined as the ratio between the standard deviation and the mean). (B) Multidimensional scaling (MDS) clustering plot of lncRNAs detected as having a leading fold change (calculated by EdgeR in RNA-Seq samples from leukocyte RNA-Seq libraries of PD patients' pre-DBS and HC). The location represent the similarity between the corresponding samples. X axis (leading fold change dimension 1) is the direction that best separated the samples, regardless of type. Y axis (dimension 2) is the best direction, uncorrelated with the first, which separates the samples. (TIF) [file pcbi.1003517.s001.tif]

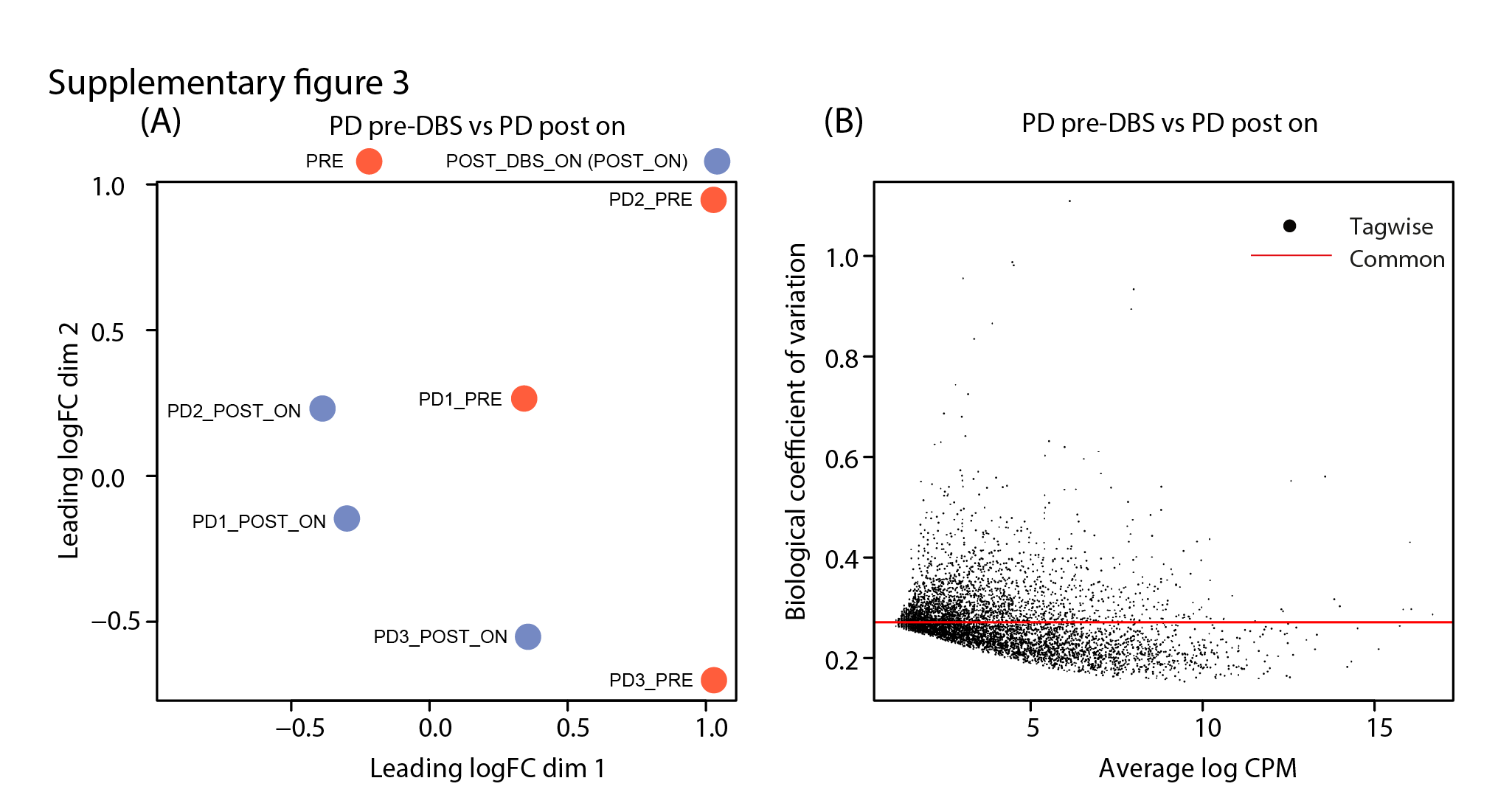

Supplement: Figure S2 — Biological Coefficient Variation (BCV) and tagwise dispersion plots for lncRNAs detected in PD brain RNA-Seq data compared to HC brain samples. (A) The BCV plot is given for the RNA-Seq samples from brain samples of PD patients and unaffected (HC) individuals (accession number E-GEOD-40710). X axis: log CPM. Y axis: the biological coefficient of variation. (B) MDS plot leading fold change of lncRNAs detected as modified (by EdgeR analysis) between PD patients pre- and post-DBS in RNA-Seq libraries from blood leukocytes RNA. The location represent the similarity between the corresponding samples. X axis: dimension 1, y axis: dimension 2. (TIF) [file pcbi.1003517.s002.tif]

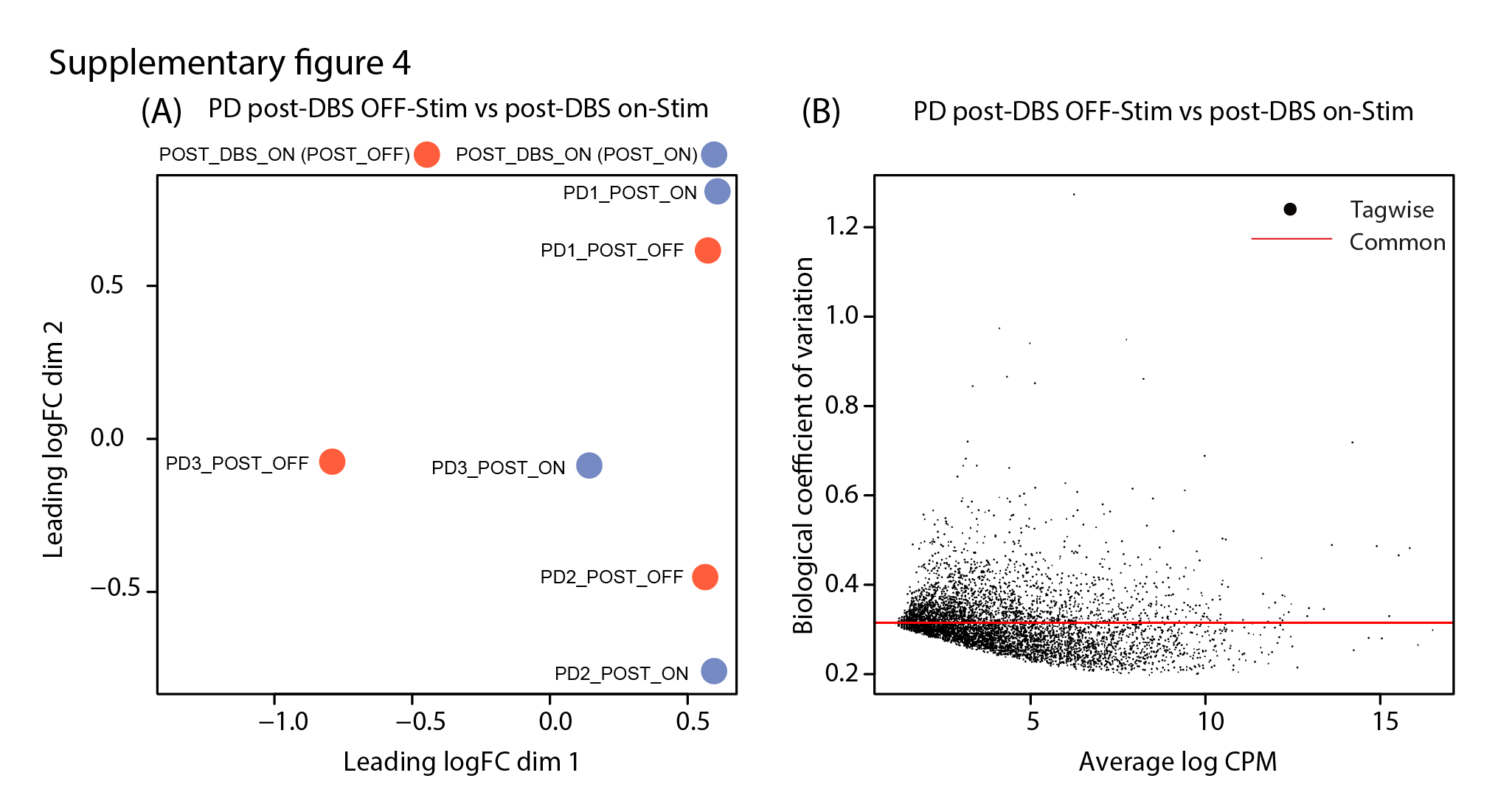

Supplement: Figure S3 — Biological Coefficient Variation (BCV) and tagwise dispersion plots for PD pre- compared to post-DBS leukocyte state of lncRNAs analyzed from RNA-Seq count reads. (A) The BCV plot is given for the RNA-Seq samples from blood leukocytes of PD patients pre- DBS and post-DBS on electrical stimulation. X axis: log CPM. Y axis: the biological coefficient of variation. (B) MDS plot leading fold change of lncRNAs detected as modified (by EdgeR analysis) between PD patients pre- and post-DBS in RNA-Seq libraries from blood leukocytes RNA. The location represent the similarity between the corresponding samples. X axis: dimension 1, y axis: dimension 2. (TIF) [file pcbi.1003517.s003.tif]

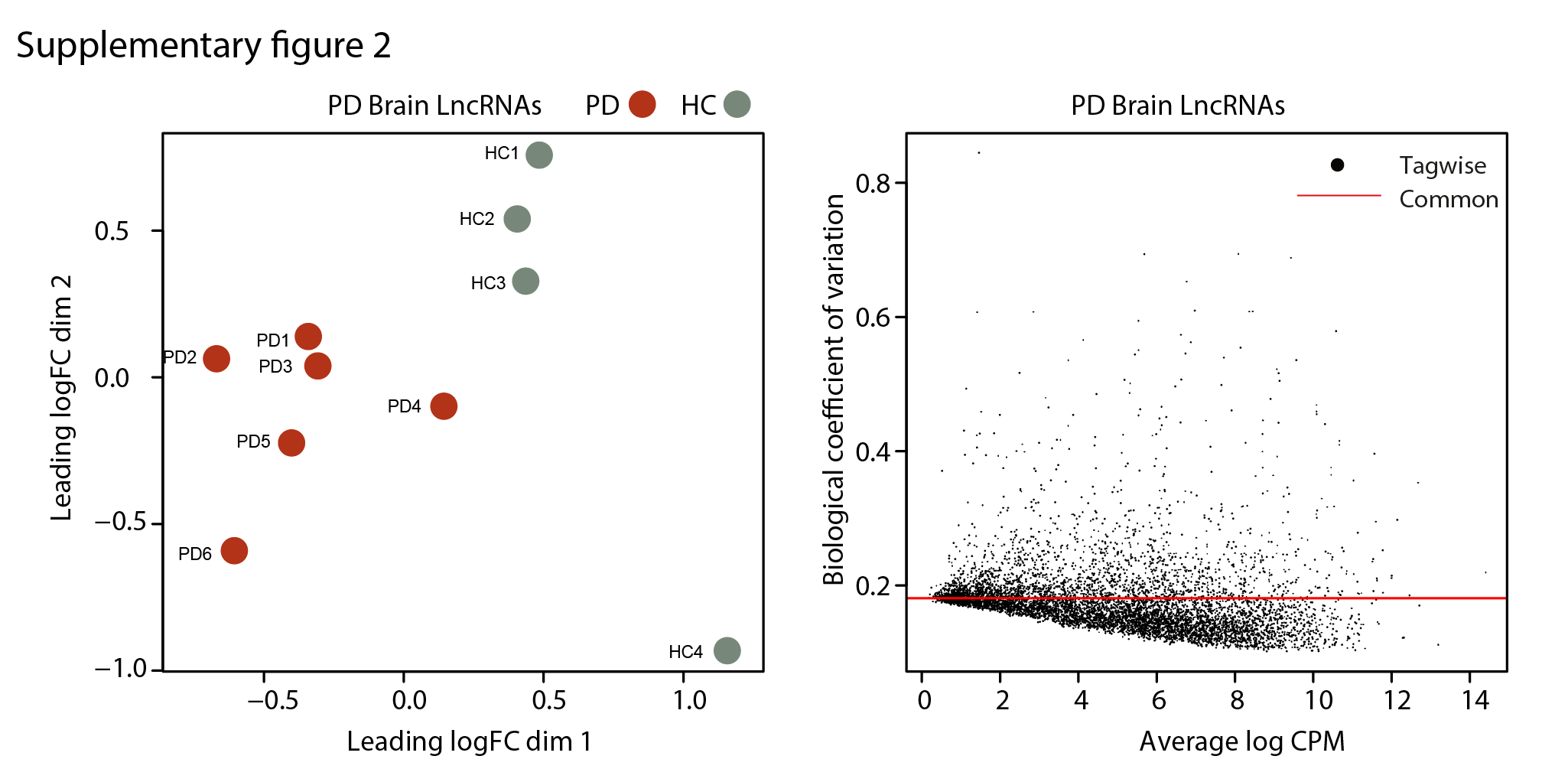

Supplement: Figure S4 — Biological Coefficient Variation (BCV) and tagwise dispersion plots for PD patients post-DBS on stimulation compared to following one hour of electrical stimulation cessation (OFF-state). (A) The BCV plot is given for the RNA-Seq samples from blood leukocytes of PD patients post-DBS on and following one hour of electrical stimulation cessation. X axis: log CPM. Y axis: the biological coefficient of variation. (B) MDS plot leading fold change of lncRNAs detected as modified (by EdgeR analysis) between PD patients post on-stimulation and following one hour off- electrical stimulation of RNA-Seq libraries from blood leukocytes RNA. The location represent the similarity between the corresponding samples. X axis: dimension 1, y axis: dimension 2. (TIF) [file pcbi.1003517.s004.tif]

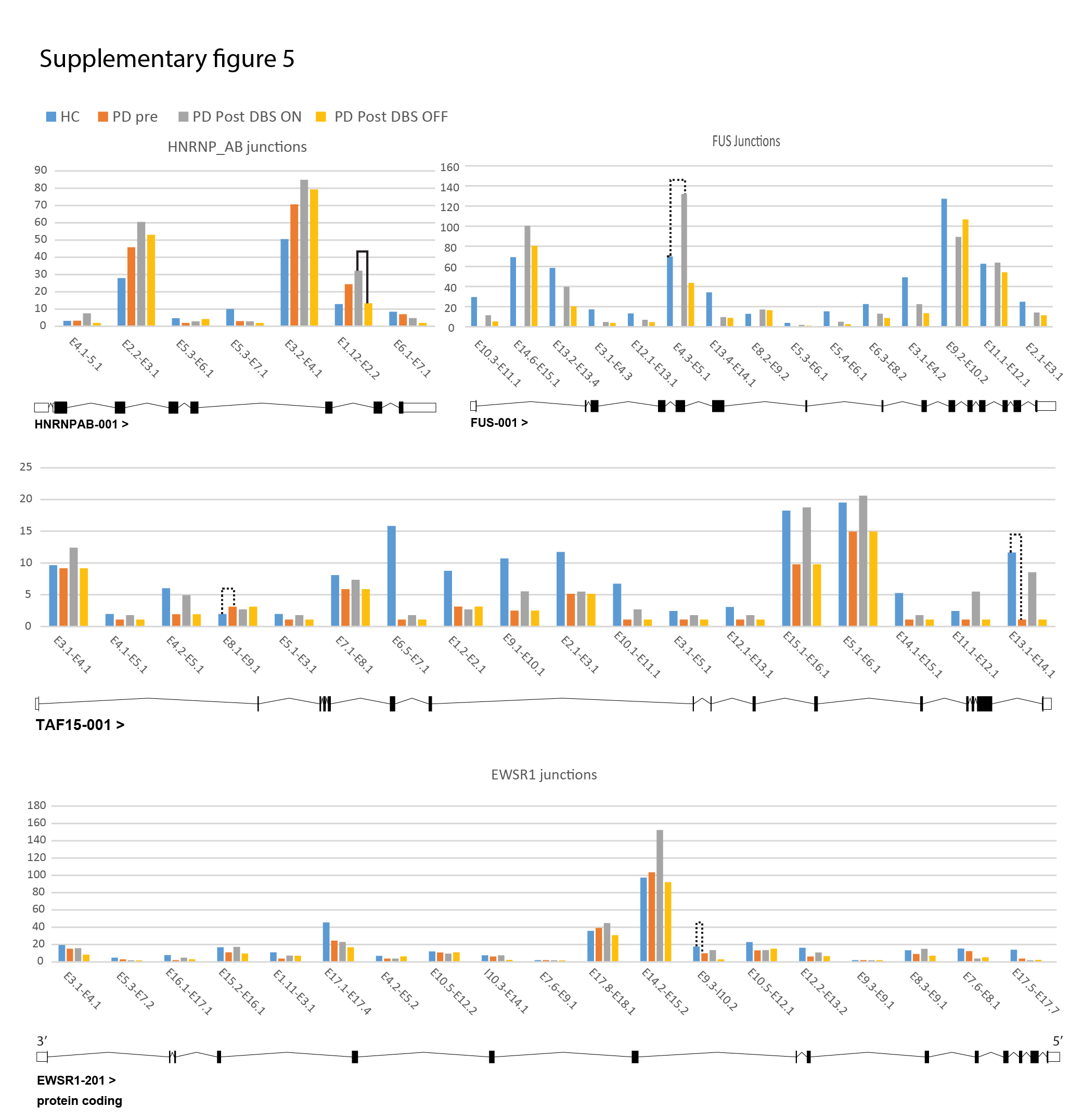

Supplement: Figure S5 — Alternatively spliced neuropathologies-related genes which include prion-like domains. Prion-like protein domains were found as enriched in previously neuropathology-related genes, including FUS, EWSR1, TAF15 and the HNRNP A/B. Gene structures include white boxes for untranslated regions, black boxes for translated exons and lines for introns. The junction probe-sets that were identified as differentially expressed are marked in solid (p<0.05) or dashed (p<0.001) lines. (TIF) [file pcbi.1003517.s005.tif]

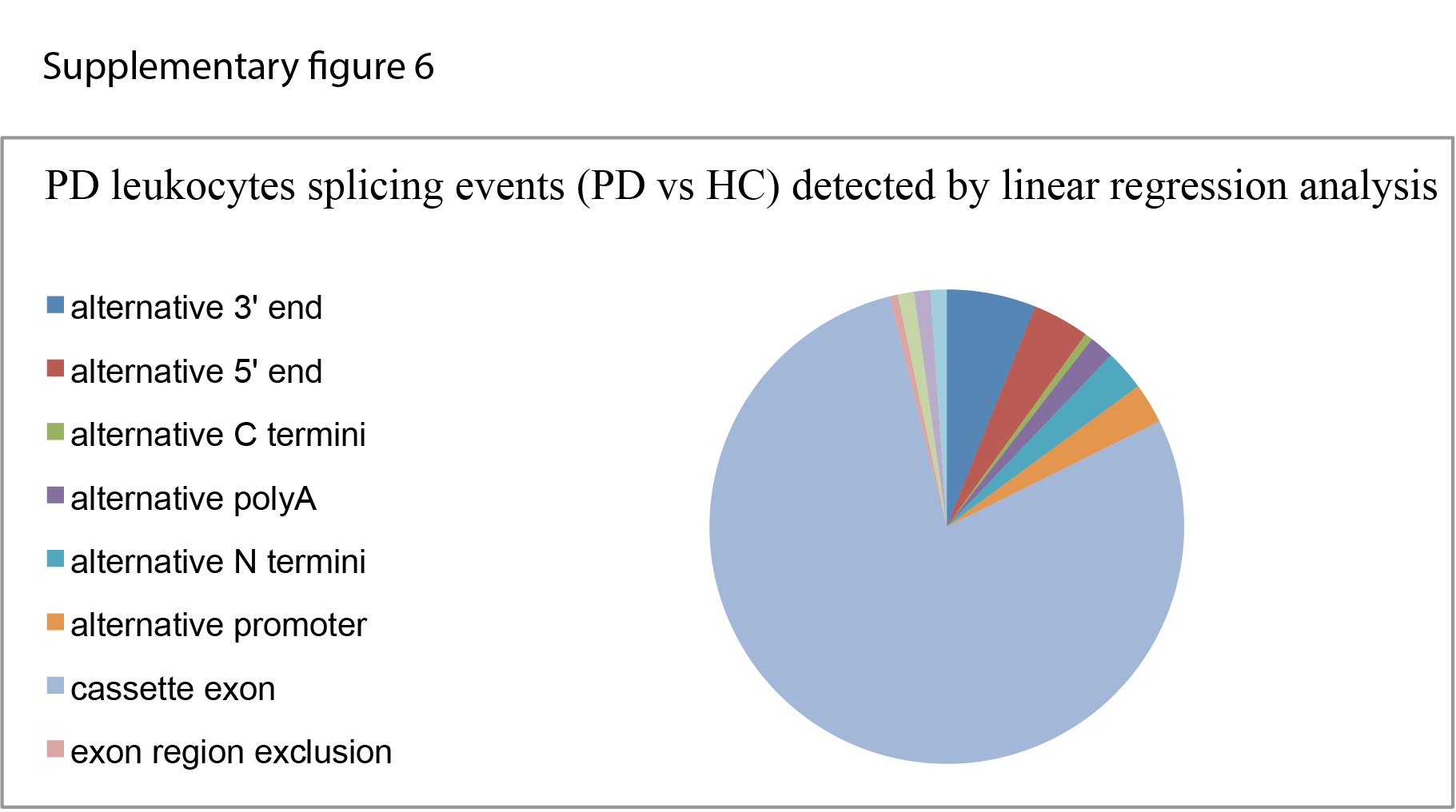

Supplement: Figure S6 — PD splicing modulated transcript regions. The distribution of different splicing event types detected in PD patients' blood leukocytes as compared with matched healthy control volunteers through linear regression analysis of all the reciprocal junction pairs expressed in the corresponding RNA-Seq libraries included cassette exons (the majority), alternative terminals, alternative promoters and alternative polyA- site usage. (TIF) [file pcbi.1003517.s006.tif]
